# Novel strains of *Culex flavivirus* and Hubei chryso-like virus 1 from the *Anopheles* mosquito in western Kenya

**DOI:** 10.1016/j.virusres.2023.199266

**Published:** 2023-11-11

**Authors:** Olivia Wesula Lwande, Jonas Näslund, Andreas Sjödin, Rebecca Lantto, Verah Nafula Luande, Göran Bucht, Clas Ahlm, Bernard Agwanda, Vincent Obanda, Magnus Evander

**Affiliations:** a Department of Clinical Microbiology, Umeå University, Umeå 901-85, Sweden; b Umeå Centre for Microbial Research, Umeå University, Umeå 901-87, Sweden; c Swedish Defence Research Agency, CBRN, Defence and Security, Umeå 901 82, Sweden; d Mammalogy Section, National Museums of Kenya, Nairobi 40658-00100, Kenya; e Department of Research Permitting and Compliance Wildlife Research and Training Institute, Naivasha 842-20117, Kenya

**Keywords:** *Culex flavivirus*, Hubei chryso-like virus 1, mosquito-borne viruses, Next generation target enrichment protocol, Western Kenya, *Anopheles spp*

## Abstract

Surveillance of mosquito vectors is critical for early detection, prevention and control of vector borne diseases. In this study we used advanced molecular tools, such as DNA barcoding in combination with novel sequencing technologies to discover new and already known viruses in genetically identified mosquito species. Mosquitoes were captured using BG sentinel traps in Western Kenya during May and July 2019, and homogenized individually before pooled into groups of ten mosquitoes. The pools and individual samples were then used for molecular analysis and to infect cell cultures. Of a total of fifty-four (54) 10-pools, thirteen (13) showed cytopathic effect (CPE) on VeroB4 cells, eighteen (18) showed CPE on C6/36 cells. Eight (8) 10-pools out of the 31 CPE positive pools showed CPE on both VeroB4 and C6/36 cells. When using reverse transcriptase polymerase chain reaction (RT-PCR), Sanger sequencing and Twist Comprehensive Viral Research Panel (CVRP) (Twist Biosciences), all pools were found negative by RT-PCR when using genus specific primers targeting *alphaviruses, orthobunyaviruses* and virus specific primers towards o'nyong-nyong virus, chikungunya virus and Sindbis virus (previously reported to circulate in the region). Interestingly, five pools were RT-PCR positive for flavivirus. Two of the RT-PCR positive pools showed CPE on both VeroB4 and C6/36 cells, two pools showed CPE on C6/36 cells alone and one pool on VeroB4 cells only. Fifty individual mosquito homogenates from the five RT-PCR positive 10-pools were analyzed further for flavivirus RNA. Of these, 19 out of the 50 individual mosquito homogenates indicated the presence of *flaviviru*s RNA. Barcoding of the *flavivirus* positive mosquitoes revealed the mosquito species as *Aedes aegypti* (1), *Mansonia uniformis* (6), *Anopheles* spp (3), *Culex pipiens* (5), *Culex spp* (1), *Coquilletidia metallica* (2) and *Culex quinquefasciatus* (1). Of the 19 flavivirus positive individual mosquitoes, five (5) virus positive homogenates were sequenced. Genome sequences of two viruses were completed. One was identified as the single-stranded RNA *Culex flavivirus* and the other as the double-stranded RNA Hubei chryso-like virus 1. Both viruses were found in the same *Anopheles* spp. homogenate extracted from a sample that showed CPE on both VeroB4 and C6/36 cells. The detection of both viruses in a single mosquito homogenate indicated coinfection. Phylogenetic analyses suggested that the *Culex flavivirus* sequence detected was closely related to a *Culex flavivirus* isolated from Uganda in 2008. All four Hubei chryso-like virus 1 segments clusters closely to Hubei chryso-like virus 1 strains isolated in Australia, China and USA. Two novel strains of insect-specific viruses in *Anopheles* mosquitoes were detected and characterized.

## Highlights

• Complete sequences of Hubei chryso-like virus segments and the genome of *Culex flavivirus* in *Anopheles* spp*.*
• *Culex flavivirus* and Hubei chryso-like virus in a single *Anopheles* spp. indicates coinfection.
• Insect specific viruses may not be mosquito-species specific.
• First report of utilisation of Twist CVRP to detect viruses from mosquito samples.

## Graphical abstract

**Figure:**
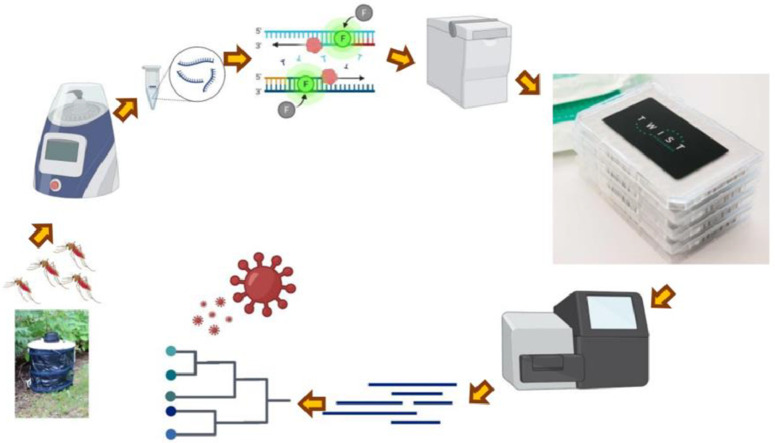

## 1 Introduction

Pathogenic mosquito-borne viruses, especially those belonging to major families such as *Togaviridae* (e.g. chikungunya virus)*, Flaviviridae* (e.g. dengue virus)*, Phenuiviridae* (e.g. Rift Valley fever virus) have been widely explored due to their significance to public and veterinary health (Baudin et al., 2016; Hassan et al., 2011; Konongoi et al., 2016; Powers and Logue, 2007; Sang and Dunster, 2001; Zeller et al., 2013). Further, the recent advancement of powerful molecular tools including the next generation sequencing (NGS) platforms/methods/machines and the development of highly specialized bioinformatic platforms, has provided means of studying mosquito virome through metagenomic analyses (Frey et al., 2016; Junglen and Drosten, 2013; Roundy et al., 2017; Shi et al., 2016). This has resulted in the discovery of novel viruses, and many of those are insect-specific viruses (ISVs), which belong to similar families as the pathogenic mosquito borne viruses (Frey et al., 2016; Junglen and Drosten, 2013; Roundy et al., 2017; Shi et al., 2016). Metagenomic analysis of virus sequences from mosquitoes has also revealed the presence of novel double stranded (ds) RNA viruses for example *Chrysoviridae* related (Hubei chryso-like virus 1), Culex Negev-like virus 3 (Biggie/Goutanap virus like) and virus related to Hubei reo-like virus 7 (Vibin et al., 2018). The implication of these viruses to public, veterinary as well as the environment is not known.

Since the discovery of the first ISVs (Cell-fusing agent virus - CFAV) about four decades ago (Stollar and Thomas, 1975), they have attracted attention, due to their potential role in biocontrol, vaccine development and contribution towards the understanding of the mechanisms of host restriction and host range, as many of them do not infect vertebrate cells (Roundy et al., 2017). ISVs are believed evolved long-ago, resulting in different lineages across diverse insect hosts, mainly mosquitoes (Cook et al., 2013). This is supported by vertical transmission studies where indications of possible integration of the virus genome into the germinative cells has been observed (Bolling et al., 2012; Cook et al., 2006; Roiz et al., 2009).

Most mosquito specific viruses belong to the family *Flaviviridae,* for example: CFAV, which was initially isolated from an *Ae. aegypti* cell line supernatant inoculated onto an *Ae. albopictus* cell line; *Culex flavivirus* (CxFV) originally isolated from *Cx. pipiens* and other *Culex* spp. in 2007 in Japan (Hoshino et al., 2007) with subsequent isolations in Guatemala, (Morales-Betoulle et al., 2008), Mexico (Farfan-Ale et al., 2009; Saiyasombat et al., 2010), the United States (Blitvich et al., 2009; Bolling et al., 2011; Crockett et al., 2012; Kim et al., 2009; Newman et al., 2011), Trinidad (Kim et al., 2009), Italy (Roiz et al., 2009), Uganda (Cook et al., 2009), Europe (Calzolari et al., 2010; Vázquez et al., 2012), China (Huanyu et al., 2012; Liang et al., 2015), Brazil (Machado et al., 2012), Taiwan (Chen et al., 2013) and Argentina (Goenaga et al., 2014); Kamiti River virus (KRV) isolated from the development stages of *Ae. mcintoshi* in 1999 in Kenya (Sang et al., 2003)and the Aedes flavivirus (AeFV) isolated from pools of *Ae. albopictus* and *Ae. flavopictus* in 2009 in Japan (Hoshino et al., 2009) with subsequent isolations in Europe (Roiz et al., 2012) and the Americas (Fernandes et al., 2016; Haddow et al., 2013). However, ISVs belonging to other virus families have also been discovered including: the *Reoviridae* family comprising of Aedes pseudoscutellaris reovirus (APRV) (Attoui et al., 2005) and Fako virus (FAKV) (Auguste et al., 2015); *Togaviridae* comprising of Eilat virus isolated from *Anopheles coustani* in Israel in 1982–1984 (Samina et al., 1986)and *Peribunyaviridae* comprising of Badu virus isolated from *Culex* spp. mosquitoes in 2003 in Australia (van-den-Hurk et al., 2008). In addition to the new virus taxon- *Negevirus* which includes six prototype ISVs i.e. Negev (NEGV), Ngewotan (NWTV), Piura (PIUV), Loreto (LORV), Dezidougou (DEZV) and Santana (SANV), isolated from mosquitoes and phlebotomine sandflies collected in Brazil, Peru, USA, Ivory Coast, Israel and Indonesia (Vasilakis et al., 2013). The first ISV that was discovered in Kenya - the KRV [35], has been followed by other ISVs, for example CxFV from *Culex quinquefasciatus* in western and coastal regions of Kenya (Iwashita et al., 2018), *Aedes flaviviruses* from *Aedes aegypti, Aedes luteocephalus, Aedes* spp. and *Cx. pipiens* at Lake Victoria and *Anopheles flavivirus* from *An. gambiae* at Lake Baringo (Ajamma et al., 2018). In addition, metagenomic analysis of Culex mosquitoes in Kwale, Kenya reveal the presence of diverse ISVs belonging to *Baculoviridae* (Atoni et al., 2018).

The current study employed an NGS target enrichment protocol specific for viruses known as the Twist Comprehensive Viral Research Panel (CVRP) (Twist Biosciences) that covers reference sequences for 3153 viruses, including 15,488 different strains. Although the method has not been applied on mosquito-borne viruses, it has been proven to be simple, reliable and accurate in screening of patient samples for infectious viral pathogens as in the case of respiratory viral co- infections with Rhino and Influenza virus in patients confirmed to have SARS-CoV-2 (Kim et al., 2021). The CVRP, has been designed to be applicable within the Illumina TruSeq RNA Library Prep for Enrichment and TruSeq RNA Enrichment workflows.

We tested whether the kit could be used to detect known and unknown viruses from mosquitoes. Therefore, we utilized the opportunity by testing randomly selected RNA extracted from mosquito samples obtained during an ongoing surveillance in western Kenya. We believe that the findings from this study may play a critical role in the discovery and detection of pathogens in vectors and hosts. Viral detection is critical to understanding the dynamics of viral populations and their interactions with vectors and hosts. This will enhance knowledge about unknown human pathogenic viruses which could be potentially used biological control of mosquitos.

## 2 Materials and methods

### 2.1 Mosquito trapping and sorting

**Fig. 1:**
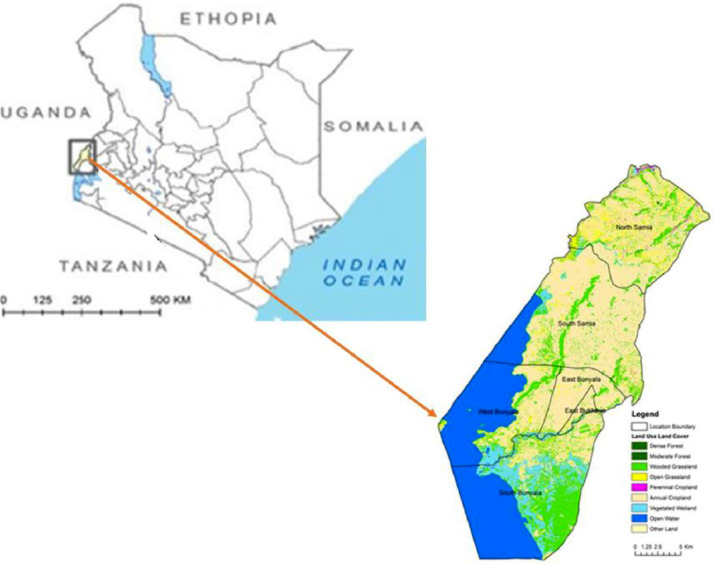
Map of Kenya showing study area in Busia County western Kenya. Inset shows the specific study sites including in Funyula and Budalangi where the mosquitoes were collected. The study area is close to Lake Victoria and the border between Kenya and Uganda. The area is known to be endemic to malaria with majority of mosquitoes being *Anopheles gambiae* the key vectors of *Plasmodium falciparum* that causes malaria. Additionally, Budalangi is prone to flooding especially during rainy seasons. The area is also highly infested by tsetseflies.

The study was conducted in Busia County, Western Kenya close to the Kenya-Uganda border (Fig. 1), and the mosquitoes were captured in Funyula and Budalangi during May and July 2019 using BG sentinel traps (Biogent, Germany). The trapping areas are heavily forested with bushy woodland which is infested with mosquitoes and tsetse flies. The areas are prone to flooding especially during long rainy seasons, mainly occuring along the Budalangi flood plain area (Lutomiah et al., 2013). The captured mosquitoes were anesthetized, sorted based on date of collection, site and stored at −80 °C, pending processing.

### 2.2 Mosquito homogenization and sampling

Each mosquito was prepared individually in a 2 mL micro tube with cap (Sarstedt, Nümbrecht, Germany) containing steel beads (2 mm diameter) (AB Nino Lab, Upplands Väsby, Sweden) and 350 μL of 1x sterile filtered Dulbecco's Modified Eagles Media (DMEM) (Sigma-Aldrich, St Louis, MO, US) with 2 % HEPES (Fisher Scientific, Fair Lawn, NJ, US). Homogenisation was performed using FastPreps 120 (Q-BIOgene, Irvine, CA, US) at 6.5 m/s for 20 s.

Sixty (60) μL of mosquito homogenates from 10 individual samples were used to create 10x pools, accordingly, adding up to a total volume of 600 μL. The process was performed at 4 °C to maintain the integrity of samples and virus viability, and subsequently stored at −80 °C.

### 2.3 Cell culture of mosquito pools

Vero B4 cells and C6/36 cells were grown in a 24 well plate to 80 % confluency in DMEM and Leibovitz media containing 10 % fetal bovine serum (FBS) (GE Healthcare Life Sciences, South Logan, UT, US) and 2 % penicillin/streptomycin (PEST) (GE Healthcare Life Sciences, South Logan, UT, US) respectively. The cells were then rinsed with sterile phosphate-buffered saline (PBS), and 100 μL of clarified 10x mosquito homogenate was added to each well (in duplicate), followed by incubation at 37 °C (Vero B4 cells) and 28 °C (C6/36 cells) for 45 min to allow virus adsorption. After incubation, 1 mL DMEM and Leibovitz media supplemented with 2 % fetal bovine serum (FBS) (GE Healthcare Life Sciences, South Logan, UT, US) and 2 % penicillin, streptomycin (PEST) (GE Healthcare Life Sciences, South Logan, UT, US) was added into the wells and the cells allowed to incubate at 37 °C (Vero B4 cells) and 28 °C (C6/36 cells) for 14 days while observing cytopathic effect (CPE) on a daily basis. The supernatants of Vero B4 and C6/36 cells exhibiting CPE of approximately 50 % were harvested from the wells by gently scraping the bottom of each well with a Pasteur pipette and transferred to 1 mL cryovials for storage at −80 °C before a further round of inoculation, as previously described.

### 2.4 RNA extraction

Extraction of viral RNA, from the pooled and individual mosquito homogenates was performed with QIAmp® Viral RNA Mini Kit (QIAGEN, Hilden, Germany), According to the manufacturer's protocol (Spin Protocol). One hundred forty (140) μL of each CPE positive 10x mosquito homogenate pool was used as a sample volume and eluted in a final volume of 60 μL, collected in 1.5 mL sterile Eppendorf tubes and stored at −80 °C.

### 2.5 cDNA synthesis, PCR, gel electrophoresis and sequencing

The extracted RNA was converted to cDNA using the Revert Aid RT kit (Thermo Fisher Scientific,Waltham, Massachusetts, US) according to manufacturer's instructions. PCR was performed using genus specific primers targeting the non-structural protein 5 (NS5) of the *flavivirus* genomes (Bryant et al., 2005). Briefly, conventional PCR was performed using the Phusion Green Hot Start II High- Fidelity PCR Master Mix (Thermo Fisher Scientific). For each reaction, 2 μL of template was used together with 10 μL of the 2x Phusion mix, 1.25 μL of both forward (FU 1; 5′- TAC AAC ATG ATG GGA AAG AGA GAG AA-3′) and reverse primers (CFD2; 5′- GTG TCC CAG CCG GCG GTG TCA TCA GC-3′) (10 pmol), 0.6 μL of DMSO and 4.9 μL of nuclease free water, up to a total reaction volume of 20 μL. Conditions for reactions were 98 °C for 30 s for initial denaturation. Further, amplification was performed using 35 cycles of: 98 °C for 7 s, 60 °C for 15 s and 72 °C for 20 s. Final extension was performed at 72 °C for 7 min. The PCR products were analysed by gel electrophoresis using 3 % agarose in 1x TAE with GelRed (Biotium Inc. Hayward, CA, US) and later purified with ExoSAP-IT kit (Thermo Fisher Scientific) and sent to Eurofin Genomics (Germany) for Sanger sequencing. Sequences were then aligned to previously identified Flavivirus strains in GenBank using the Basic Local Alignment Search Tool (BLAST) provided by the National Center for Biotechnology Information.

### 2.6 DNA barcoding of mosquito species

Approximately 50 μL of the individual Flavivirus positive mosquito homogenates were used for DNA extraction using NucleoSpin® DNA Insect (Macherey-Nagel, Düren, Germany) according to the manufacturer's instructions. The DNA was stored at −80 °C. Amplification of extracted DNA was performed using Phusion Green Hot Start II High-Fidelity PCR Master Mix (Thermo Fisher Scientific,) with a LCO/HCO primer pair, targeting the mitochondrial cytochrome c oxidase subunit I gene (COI) (Folmer et al., 1994). For each reaction, 2 μL of template was used together with 10 μL of 2x Phusion mix, 1.25 μL of both forward and reverse primers (10 pmol), 0.6 μL of DMSO and 4.9 μL of nuclease free water, up to a total reaction volume of 20 μL. Conditions for reactions were 98 °C for 30 s for initial denaturation. Further, amplification was performed using 35 cycles of: 98 °C for 7 s, 50 °C for 15 s and 72 °C for 20 s. Final extension was performed at 72 °C for 7 min. PCR product was analysed by gel electrophoresis using 1.2 % agarose in 1x TAE with GelRed (Biotium Inc. Hayward, CA, US) and later purified with ExoSAP-IT kit (Thermo Fisher Scientific,) and sent to Eurofin Genomics (Germany) for Sanger sequencing. Sequences were then aligned to previously identified mosquito species in GenBank using the Basic Local Alignment Search Tool (BLAST) provided by National center for Biotechnology Information.

### 2.7 Pan-Viral panel protocol

The study employed a next-generation sequencing target enrichment protocol specific for viruses known as the Twist Comprehensive Viral Research Panel (CVRP) (Twist Biosciences, San Francisco, CA, USA), which covers reference sequences for 3153 viruses, including 15,488 different strains (Kim et al., 2021). The RNA was converted to cDNA using ProtoScript II First Strand cDNA Synthesis Kit (E6560S) and New England Biolab's Random Primer 6 (S1230S). The NEBNext Ultra II Non-Directional RNA Second Strand Synthesis kit (E6111S) was subsequently used to convert single-stranded cDNA to dsDNA. Next, DNA fragmentation, End repair, and dA-Tailing were performed. Universal Twist adapters were then ligated to the dA-tailed DNA fragments and purified to generate cDNA fragments libraries ready for indexing through amplification. Finally, PCR amplification of the adapted cDNA libraries with Twist UDI primers, purification and quality control was conducted to index the samples and library preparation finalized. A single pooled library (9.6 ng/μL) was first prepared from the indexed library-prepped sample pools. This was followed by hybridization of the targets in solution, which was ∼16 h in total to complete. Thereafter, the binding of hybridized targets to desired streptavidin beads was performed. Libraries were then enriched *via* PCR amplification and purification utilizing 23 cycles as recommended by Twist Technical Support. Sample libraries were sequenced with 75 bp paired-end reads on the Illumina MiSeq platform, using a MiSeq Reagent v3 150-cycles kit according to manufacturer protocols. Sequencing data was processed according to methods described below.

### 2.8 Taxonomic classification of metagenomic reads

Generated sequence reads were initially depleted for potential host reads by mapping to human reference (GRCh37) and mosquito species (*Aedes aegypti* strain LVP_AGWG and *Culex quinquefasciatus* strain JHB). Remaining sequence reads were classified using Kaiju (Menzel et al., 2016) to give a profile of potential virus species in enriched samples.

### 2.9 Virus genome assembly, coverage analysis and variant detection

Depleted sequence reads were assemblies using Megahit (Li et al., 2015) and Trinity (Grabherr et al., 2011) and contigs longer than 1000 bp were kept and polished using Pilon (Walker et al., 2014). Remaining contigs were annotated using Prokka (Seemann, 2014) and characterized using Checkv (Nayfach et al., 2021) and Virsorter (Roux et al., 2015). Predicted virus sequences were then further annotated and confirmed using NCBI Blast.

### 2.10 Amino acid substitution and phylogenetic analyses

The sequences obtained from the study (accession numbers OK413943, OK413944 OK413945 OK413946 [Hubei chryso-like virus segment 1 to 4, respectively] and OK413947 [CxFV]) segments were initially screened against ICTV species representatives using mash (Ondov et al., 2019). Similar segments were extracted from NCBI using BLAST against NT database. Segments showing higher than 50 % similarities and coverage higher than 50 % ware aligned to the respective virus sequences using MAFFT (Katoh and Standley, 2013). Phylogenetic trees were constructed from nucleotide alignments using the Maximum Likelihood method implemented in IQ-TREE 2 (Minh et al., 2020).

## 3 Results

### 3.1 Mosquito screening and virus isolation in Vero B4 and/or C6/36 cells

A total of 540 mosquitoes were homogenized and processed into 54 pools (10 mosquitoes/pool). Of the 54 pools, thirteen (13) pools showed cytopathic effect (CPE) on VeroB4 cells whereas 18 pools showed CPE on C6/36 cells translating to a total of 31 CPE positive pools. Eight (8) of the 31 CPE positive pools showed CPE on both VeroB4 and C6/36 cells (see supplementary Table 1).

### 3.2 Reverse transcriptase polymerase chain reaction RT-PCR

**Fig. 2:**
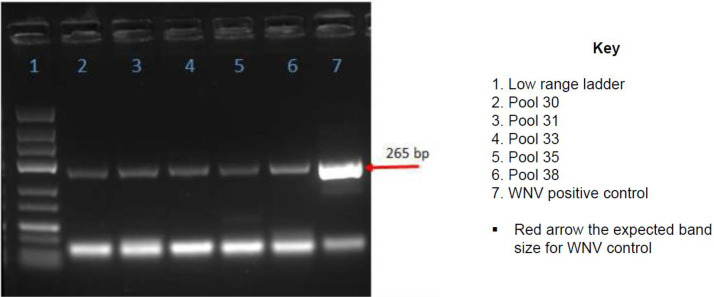
Gel photo of the five pools designated; 30, 31, 33, 35, 38 that were flavivirus positive *via* RT-PCR. The expected band size was 265 bp. All the samples had similar band size as the positive control.

**Fig. 3:**
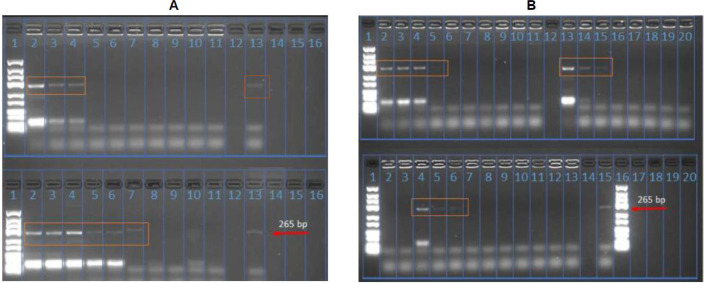
Gel photo of the 19 out of the 50 individual mosquito homogenates that constituted the five flavivirus positive pools were found to be flavivirus positive by RT-PCR (Fig. 3). Red arrow the expected band size for WNV control 265 bp. The orange text boxes show the positive individual bands.

All the 54 pools including those that were CPE positive and negative were negative by RT-PCR using genus specific primers targeting *alphavirus* and *orthobunyavirus*. The pools were also negative for specific viruses including, o'nyong-nyong virus, chikungunya virus and Sindbis virus. However, five pools designated; 30, 31, 33, 35, 38 (Fig. 2) were *flavivirus* positive *via* RT-PCR. Two of the flavivirus pools (30 and 35) showed CPE on both VeroB4 and C6/36 cells, two additional pools on C6/36 cells only (31 and 38) and one pool (33) on VeroB4 cells only (Supplementary Table 1). Nineteen (19) out of the 50 individual mosquito homogenates that constituted the five *flavivirus* positive pools were found to be *flavivirus* positive by RT-PCR (Fig. 3).

### 3.3 DNA barcoding

Barcoding results of the 19 *flavivirus* positive mosquitoes revealed seven different species, including *Aedes aegypti* (1), *Mansonia uniformis* (6), *Anopheles* spp. (3), *Culex pipiens* (5), *Culex* spp (1), *Coquilletidia metallica* (2) and *Culex quinquefasciatus* (1) (Supplementary Table 2).

### 3.4 Virus detection using the sequencing next-generation sequencing target pan-viral panel - Twist Comprehensive Viral Research Panel (CVRP)

We had resources to investigate 5 of the 19 mosquitoes with the Twist CVRP pan- viral hybrid-capture panel and we selected 2 mosquito homogenates that showed CPE in both C6/36 and VeroB4 cells, 2 in C6/36 cells only and 1 in VeroB4 cells only.

One of the five individual mosquitoes subjected to the Twist CVRP hybrid-capture yielded two complete virus genome sequences. One was a CxFV encoding a polyprotein, and the other was four complete segments of the double stranded RNA virus Hubei chryso-like virus 1. Barcoding results indicated that both viruses were isolated from an *Anopheles* spp. mosquito.

### 3.5 Phylogenetic analyses

**Fig. 4:**
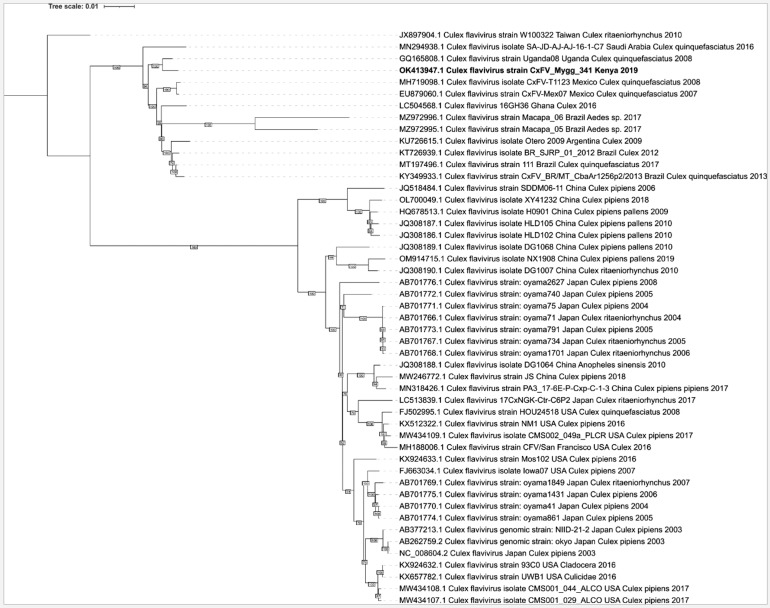
Phylogenetic analysis of complete genomes CxFV available in GenBank including the study isolate (in bold). Statistical inferences imply that the study isolate was closely related to the Ugandan strain (accession number GQ165808) detected from *Culex quinquefasciatus*. The sequences comprise the entire polyprotein gene.

Phylogenetic analysis of the detected CxFV indicated that it was closely related to the Ugandan strain isolated from *Cx. quinquefasciatus* in Uganda in 2008 (Fig. 4 and supplementary Fig. 5). All the Hubei chryso-like virus 1 segments clustered evenly with their respective virus segments from similar Hubei chryso-like virus 1 detected in Australia, China and USA (supplementary Figs. 1–4).

## 4 Discussion

The study findings revealed the presence of two different viruses, CxFV and Hubei Chryso-like virus 1 from an individual *Anopheles* spp. mosquito, sampled in western Kenya*.* The findings implied that *Anopheles* mosquitoes may play a role in the transmission and maintenance of these viruses in nature. The findings may provide insights in the ISV-mosquito interaction, as one could hypothesize that ISVs may not be mosquito-species specific. For example, it is evident that the detection of a majority of CxFV has initially been from *Culex* species of mosquitoes including; *Culex* spp in Brazil (Machado et al., 2012), *Culex quinquefasciatus* in Brazil (Moraes et al., 2019)*, Culex pipiens* in China (Fang et al., 2018) . However, the virus has now been shown to break the *Culex* mosquito species barrier and now detected in other species for instance in the *Anopheles sinensis* in China (Liang et al., 2015) and in *Anopheles* spp in the current study

Hubei chryso-like virus 1 is a relatively unknown virus, and ICTV has recently reclassified it to belong to *Alphachrysovirus shuangaoense*. These viruses are double-stranded RNA viruses, and the presence of both a single-stranded RNA virus (CxFV) and the double-stranded RNA Hubei chryso-like virus 1 in a single mosquito could potentially influence antiviral RNA interference (Keene et al., 2004; McFarlane et al., 2020; Myles et al., 2008).

Hubei chryso-like virus was originally detected in *Culex* mosquitoes in China and Australia (Shi et al., 2017; Williams et al., 2020). To our knowledge this is the first isolation of four complete segments of Hubei-Chryso-like virus in the *Anopheles* spp*.* It is clear that for many invertebrates, infection by multiple RNA viruses is likely to be the norm rather than the exception (Shi et al., 2017). However, whether the co-infection of either viruses enhances or antagonizes vector competence of the infected mosquito needs to be investigated. Previous studies have pointed to the ability of ISVs in reducing the transmission potential of pathogenic viruses such as chikungunya virus, dengue virus, West Nile virus and Zika virus (Nasar et al., 2015; Romo et al., 2018). In general, the role of other mosquito species in the transmission of ISVs should be explored considering their possible role as biological control agents and serve as a basis for arbovirus protein expression through generation of ISVs/Arbovirus chimeras.

The study also draws attention to the application and usefulness of Twist CVRP in the discovery of ISVs. To our knowledge, this is the first time the method has been utilised to detect viruses from mosquito samples. So far, the method has been deemed valid in screening of patient samples and asymptomatic health care personnel for SARS-CoV-2 (Lythgoe et al., 2021) and bat samples in Sweden where an *Alphacoronavirus* was detected in a Daubenton's Myotis bat (*Myotis daubentonii*) (Lwande et al., 2022). The fact that the method was able to generate the sequence of the entire CxFV genome and all four segments of the Hubei chryso-like virus 1 demonstrated its efficiency and robustness. Therefore, we believe that the Twist CVRP offers a platform that could aid in solving challenges emanating from the inability to detect unknown viral pathogens. There are limitations, such as the technique is based on detection of about 4000 human pathogenic viruses, thus, unrelated, unknown viruses could be missed. However, the technique is suitable for screening biological samples, especially as it can enrich 50 % of virus reads. It can also be used for characterization of semi-known viruses. We recommend that preliminary screening using genus specific primers and cell culture be used prior to selecting the samples of interest. Alternatively, explore the application of truseq of the total RNA, aiming at discovery of new viruses.

One limitation of the study was the inability of the Twist CVRP to be able to detect viruses in the remaining four CPE positive flavivirus PCR positive samples. The mosquito homogenates were free-thawed at least thrice prior to library preparation and this may have interfered with the titre which may have been already low from the start. To solve the challenge in future analysis RNA extraction and subsequent double-stranded cDNA from the same samples need to be performed the same day and aliquoted in multiple separate tubes should be generated to avoid freeze–thaw which may affect the sample integrity. We also believe that despite target enrichment other parameters like scalability, detection limit as well as reproducibility should be factored in the assay as a test of its robustness.

## 5 Conclusions

The findings implied that Twist CVRP hybrid-capture may be a robust method that could be applied for the direct detection of ISVs and other viruses vectored by mosquitoes. Moreover, the findings contributed to the much-needed genetic data, especially for under-represented dsRNA viruses like Hubei chryso-like virus 1.

## Ethics approval and consent to participate

Not applicable

## Consent for publication

Not applicable.

## Availability of data and materials

The datasets supporting the conclusions regarding sequences in this article will be available in GenBank (https://www.ncbi.nlm.nih.gov/genbank/) with accession numbers OK413943- OK413947.

## Funding

The project was supported by the Swedish Research Council grant 2017-05607 (Magnus Evander).

## CRediT authorship contribution statement

**Olivia Wesula Lwande:** Writing – original draft, Writing – review & editing, Conceptualization, Supervision, Formal analysis, Data curation. **Jonas Näslund:** Conceptualization, Formal analysis, Data curation. **Andreas Sjödin:** Formal analysis. **Rebecca Lantto:** Supervision. **Verah Nafula Luande:** Writing – original draft, Writing – review & editing, Supervision, Data curation. **Göran Bucht:** Conceptualization. **Clas Ahlm:** Writing – original draft, Writing – review & editing, Conceptualization. **Bernard Agwanda:** Conceptualization, Methodology. **Vincent Obanda:** Conceptualization, Methodology. **Magnus Evander:** Writing – original draft, Writing – review & editing, Conceptualization.

## Declaration of Competing Interest

The authors declare no competing interests.

## Data Availability

Data will be made available on request.

## Appendix Supplementary materials

Image, application 1
