# Supplementary material for: Novel strains of Culex flavivirus and Hubei chryso-like virus 1 from the Anopheles mosquito in western Kenya
Source: Virus Res. 2023 Nov 11;339:199266. doi: 10.1016/j.virusres.2023.199266 (PMC10682293; doi:10.1016/j.virusres.2023.199266)
Supplement: Supplementary file 1 [file mmc1.docx]

**Supplementary figure 1:** Phylogenetic analysis of complete genome segments of *Alphachrysovirus shuangaoense* including Hubei chryso-like virus 1 including the study isolate segments 1 (in bold). *Alphachrysovirus penicillii* was used as an outgroup. Segment 1 comprises of the RNA-dependent RNA polymerase gene

**Supplementary figure 2:** Phylogenetic analysis of complete genome segments of *Alphachrysovirus shuangaoense* including Hubei chryso-like virus 1 available in GenBank including the study isolate segments 2 (in bold). *Alphachrysovirus penicillii* was used as an outgroup. Segment 2 comprises of the putative protease gene.

**Supplementary figure 3:** Phylogenetic analysis of complete genome segments of *Alphachrysovirus shuangaoense* including Hubei chryso-like virus 1 available in GenBank including the study isolate segments 3 (in bold). *Alphachrysovirus penicillii* was used as an outgroup. Segment 3 hypothetical protein gene.

**Supplementary figure 4:** Phylogenetic analysis of complete genome segments of *Alphachrysovirus shuangaoense* including Hubei chryso-like virus 1 available in GenBank including the study isolate segments 4 (in bold). *Alphachrysovirus penicillii* was used as an outgroup. Segment 4 comprises of the hypothetical protein gene.

**Supplementary figure 5**: Phylogenetic analysis of additional complete genomes CxFV available in GenBank including the study isolate (in bold).

**Supplementary figure 6**: Genetic information of *Culex flavivirus* polyprotein gene.

**Supplementary figure 7**: Genetic information of Hubei chryso-like virus 1 segment 1 containing the RNA-dependent RNA polymerase gene

**Supplementary figure 8:** Genetic information of Hubei chryso-like virus 1 segment 2 containing the putative protease gene

# Supplementary figure 9: Genetic information of Hubei chryso-like virus 1  segment 3 containing the hypothetical protein gene

# Supplementary figure 10: Genetic information of Hubei chryso-like virus 1 segment 4 containing the hypothetical protein gene

**FASTA sequences for *Culex flavivirus* and Hubei chryso-like virus 1 segments 1, 2, 3 and 4 respectively.**

**Supplementary figure 1**


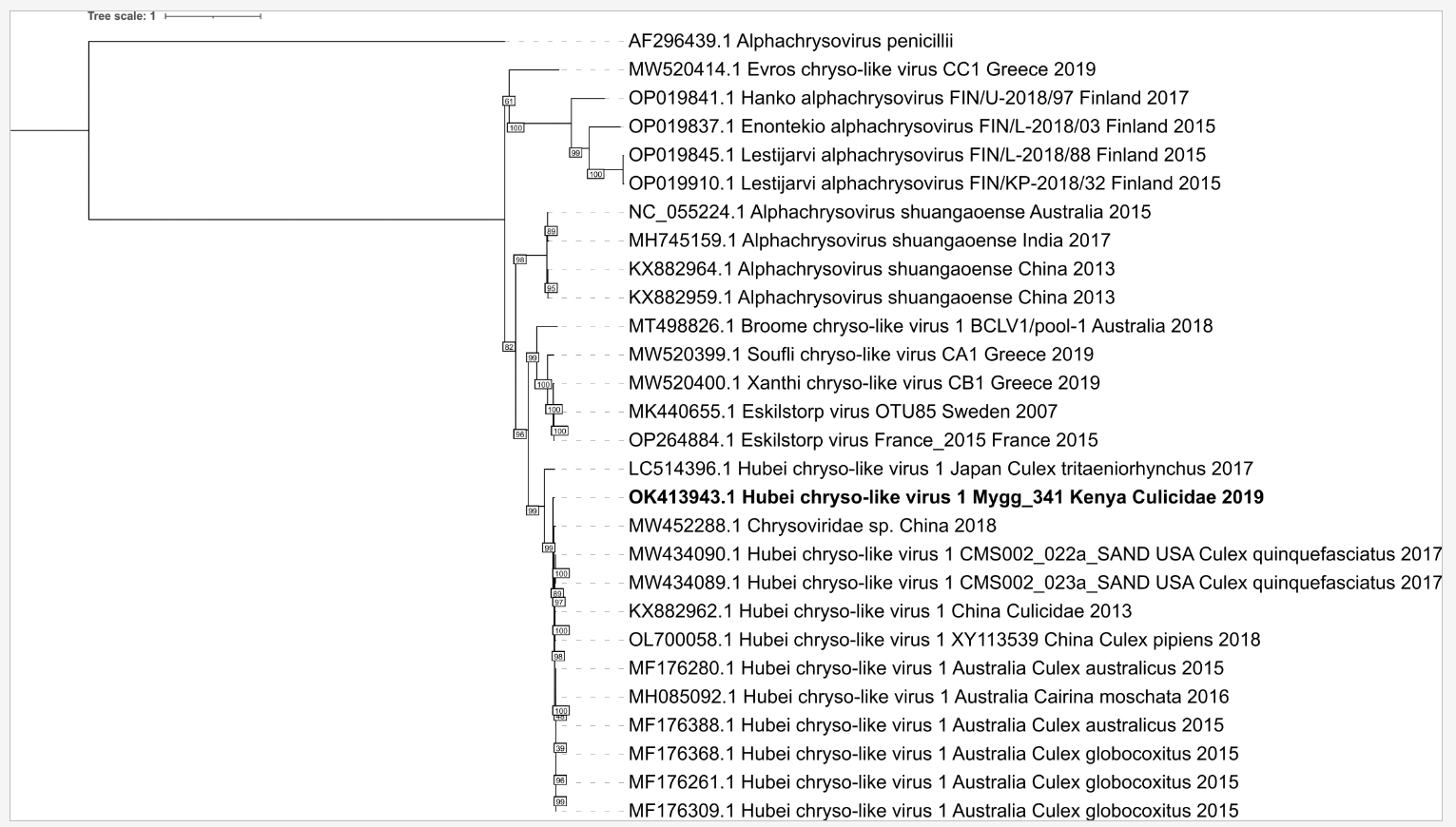


**Supplementary figure 2**


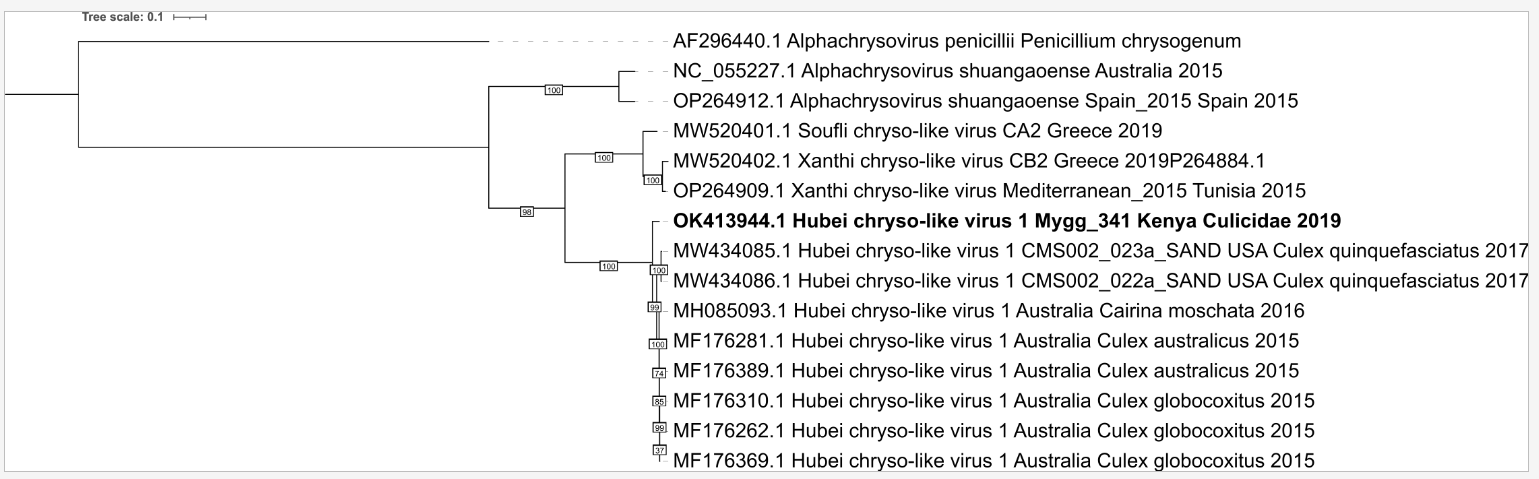


**Supplementary figure 3**


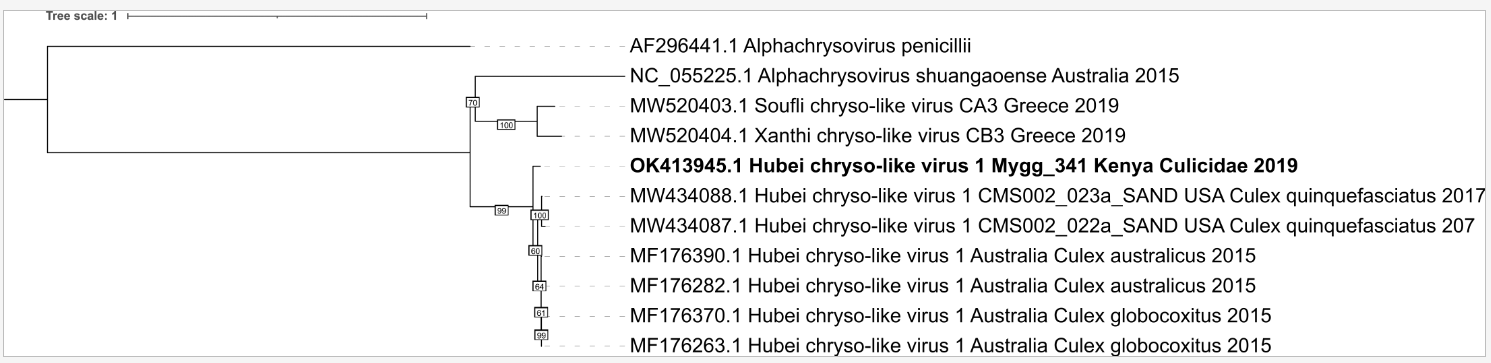


**Supplementary figure 4**


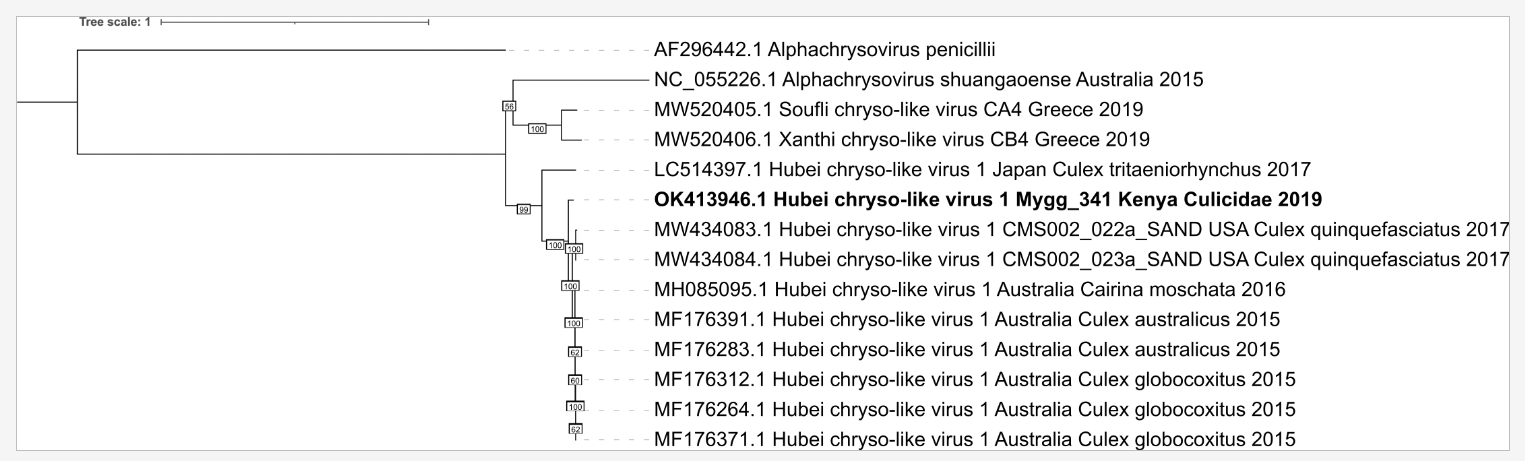


**Supplementary figure 5**


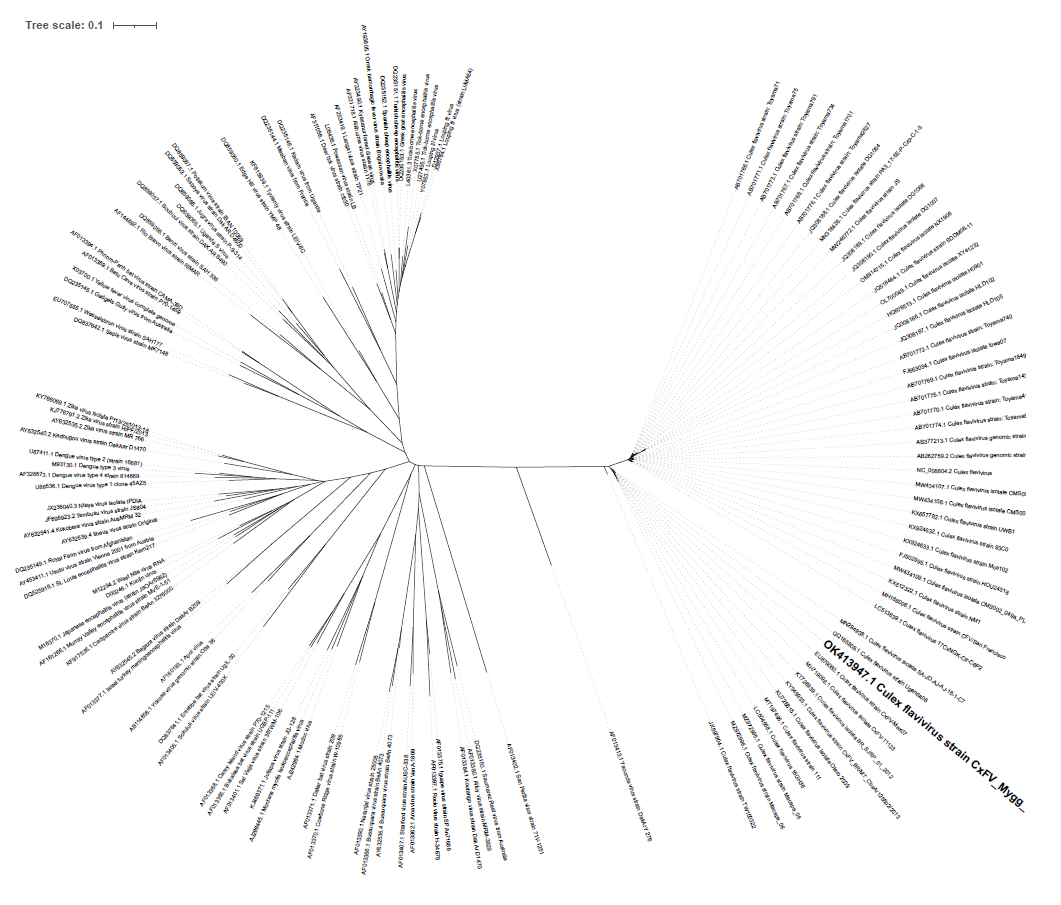


**Supplementary figure 6**


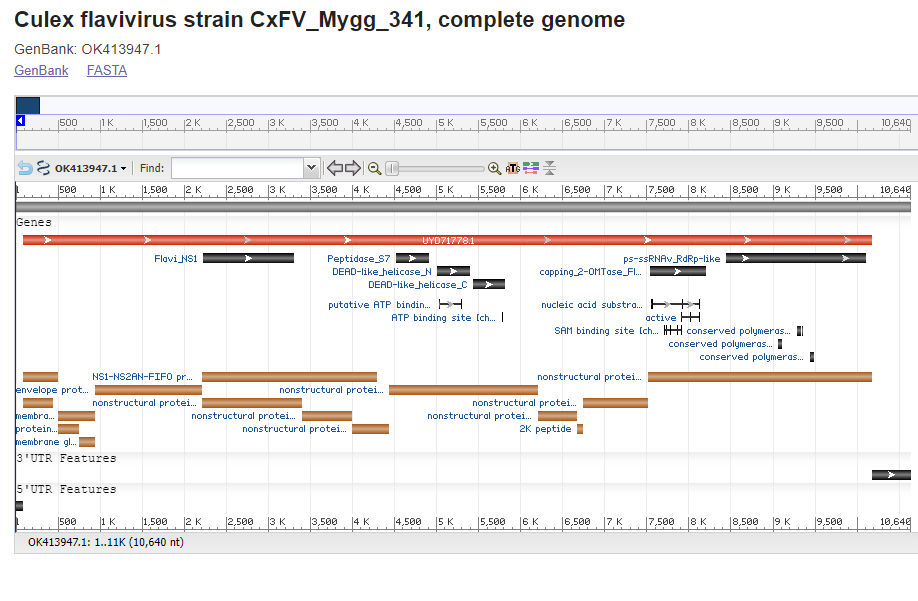


**Supplementary figure 7**


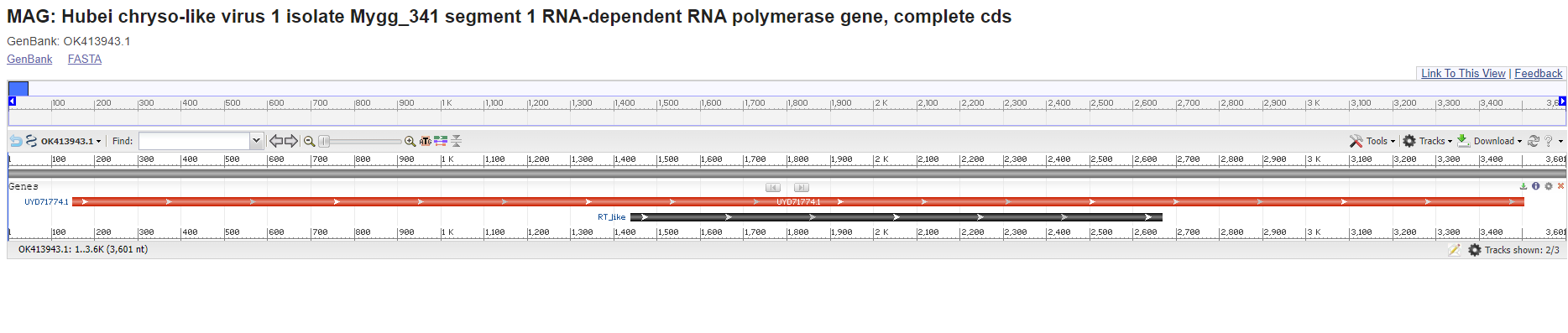


**Supplementary figure 8**


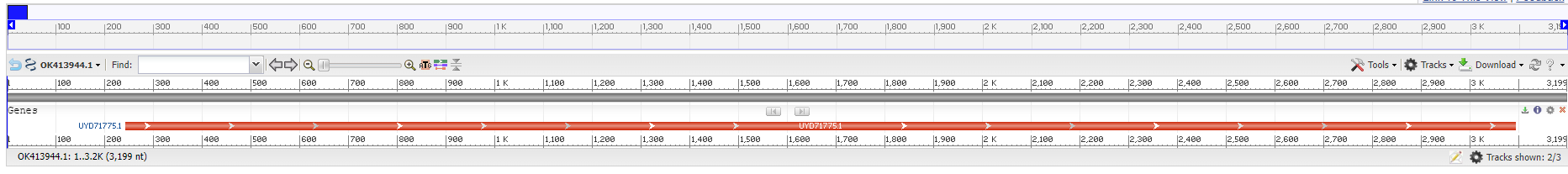


# Supplementary figure 9


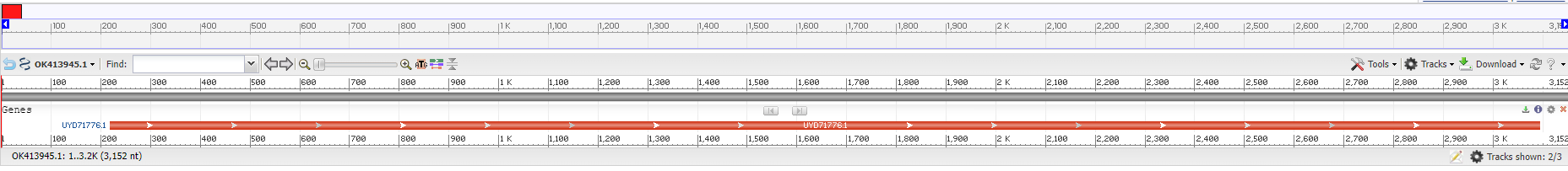


# Supplementary figure 10


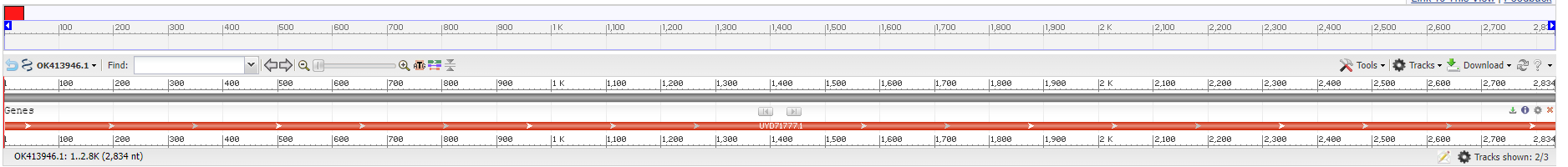


**Supplementary table 1. Mosquito homogenates pools (10x) that showed CPE in cells.**

| **Pools showing CPE in Vero B4 cells** | **Pools showing CPE in c6/36 cells** | **Pools showing CPE in both VeroB4 and c6/36 cells** |
| --- | --- | --- |
| 3 | 1 | 5 |
| 5 | 5 | 23 |
| 11 | 13 | 24 |
| 12 | 15 | 25 |
| 23 | 22 | 30 |
| 24 | 23 | 35 |
| 25 | 24 | 39 |
| 30 | 25 | 44 |
| 32 | 26 |  |
| 33 | 30 |  |
| 35 | 31 |  |
| 42 | 34 |  |
| 44 | 35 |  |
|  | 38 |  |
|  | 39 |  |
|  | 44 |  |
|  | 40 |  |
|  | 51 |  |

Red highlight indicates the 5 flavivirus positive pools. Two pools (30 and 35) showed CPE in both VeroB4 and c6/36 cells, two pools (31 and 38) showed CPE in c6/36 cells only and 1 pool (33) showed CPE on veroB4 cells only.

|  | **Mosquito sample identification** |  | **Mosquito species** |
| --- | --- | --- | --- |
| 1 | 323 |  | *Aedes aegypti* |
| 2 | 303 |  | *Mansonia uniformis* |
| 3 | 341 |  | *Anopheles costai* |
| 4 | 342 |  | *Cx. pipiens* |
| 5 | 343 |  | *Anopheles costai* |
| 6 | 291 |  | *Anopheles costai* |
| 7 | 302 |  | *Cx. pipiens* |
| 8 | 293 |  | *Mansonia uniformis* |
| 9 | 321 |  | *Cx. quinquefasciatus* |
| 10 | 375 |  | *Mansonia uniformis* |
| 11 | 292 |  | *Cx. pipiens* |
| 12 | 371 |  | *Coquillettidia metallica* |
| 13 | 322 |  | *Cx. pipiens* |
| 14 | 374 |  | *Cx. spp* |
| 15 | 372 |  | *Mansonia uniformis* |
| 16 | 301 |  | *Cx. pipiens* |
| 17 | 304 |  | *Mansonia uniformis* |
| 18 | 373 |  | *Mansonia uniformis* |
| 19 | 376 |  | *Coquillettidia metallica* |

**Supplementary table 2**. Barcoding results of the nineteen individual mosquito homogenates that gave flavivirus hits.

**FASTA sequences for for *Culex flavivirus* and Hubei chryso-like virus 1 segments 1, 2, 3 and 4 respectively.**

**>OK413947.1 Culex flavivirus strain CxFV_Mygg_341, complete genome**

TTTTTAAAAACTTTGGCTTGGTTACACCGCTGATTGGTTACACCTACATAAGGCTTGAGTTGTTTATAAT

AGTCGTTTTTCTCGCAAAGATGGGAAAGGACGACGGTAAGAAGAAGAAGGGGCCAGGCTCCTCCAGGTGG

TTGCTACCATCGGAGCGCGCTGGCTTAGGAAGGAAGGAGGAGAAAAAGAAGAAGGAGAAGAGAAGTGTGC

GGAGCACACCTCAGCTAGTCTCCGGCGGTGCGCAGCATCGTCGCGGAGGCGGAACGGGTCCTAGGGCCAG

AGGTCTGCTAGGACGCTTGGGAATTGGTTGGGGATCGATTCTCCAGGAGGACATTGTGCAGGCCCTCATG

CACTTGGTGCTGGTGCTGCACTCTGTTTTCATCGCAATTGACCGCCGCTTACGCTCACTGACACGACGCG

TGACTGCCCTTGAGGCTAAACGCTCCGCGAAGAACGCTGTACGAATCACGCTCATACTCACTGGCTTGAT

GATGGTGCTCGGCGCCGTCGTCATTGACATGCAGGTTTCCACGGCGAAAGGGACGCAGATTTTCGAGGGG

AAGACCAATCGCACCGATCATGTGCATTTGTTCAAGCTTCCCACGGATGGCTGCTGGAGTGGCACATTGG

TGATGAAAAAGTGCCCCAAGGTTGCTGATCTTGCGAAAGATCTTGAAGGAGTGGATTGTGGTTCATCATG

GACGGAATTCACTCTGCGTTACCACCGATGTGCGCCGAAGAAGCGTGAGCGACGAGCTGCCAACACCAAC

CAGAAGCTAGACTTTCTAGCAGAGGTGGAACTGGTGACGTTCAAGGCCATAAGGGAGAACAAGACCATCG

CTATCATCGTCTTGTTGTGCGTGGCCATTGCGAAGAGGTGGCCGATGTGGGTGGTAATACTACTTGCAAT

TGGAACGTGGACAACGGTGAAGGGAGAATTTGTTGAGCCGTTATACACGCTGAAGGCGGAAAAGATGACC

ATGCTGCAGACAATCATGCGACCAGACGAAAGCTACGTGATTTCCACGCCGAACGGACTTCTTGAGTTCC

GCACTGGAGCAGCCGAGATATATGGCGGCCAATGGATGCGTGAGTTGCTCGCTGATTGTCACGCAAATGC

GTCTTATTCGACCGATGTGTGTCCCGGAGGATCGCAGTTGAACATGGGTGAGATCAATGGCAAAGAACGC

GTTTGCTCAACCCAGCCCTACAATCGTGGATGGGGAACTGGATGTTTCAAGTGGGGAATCGGGTTTGTTG

CCACTTGTGTTGAACTGCATTGCGAAGAAGGTTACAATGTCTCGTCAATCGCTAGGTCATCCATCGTGAT

GAACATCACAGCGAAGTTCCACTCAGTGGATGACGTCCAGCAACTCATCAGTGATGTTCCTGTGACTTTT

CGGTTTGCAAAACTGGGCAATGCCGCTGTCACTTGCCGGCTGGAATCTGAGCGATTGCTTCTGGACTATT

ACCATGTGACGGGCAAATACCATGAAGGACTGTTTGTGCGGTCGCAAATCGATGAGTGGCCTGGAGCGCA

CGCCACTGCGAGTGGAAGAGCTGGTTTGGAACGAATCGTGGTTTGGGGAGACGCCAGGGCTAATGAAGTT

CTTGTGAAGAACATCCTGGAACCTCAGCTCATATGGGACAACGCTATTGCCACTCAAGACGGTTTTCGTG

ATGTTGGCTTCTCTTGCCAGATCATGCTTGACAAGCTGGTTAGCGGCAGTTTCAAGGATTGTCCGGGGAT

CAAATCTTCGGTGTTTGTCCAAAGTGGTTTCGGCTATGATGGAGTGGTGATGACGAGTTTGTCCAAGGCA

ACTAACGAAAGCTGCTCGGTTGGGTTGACGTGCCATGGATGCTACCTCCTCGCGACGAAGATGGTGTTCG

GACCCGGAACTTCGACTGCAAAGGCTTTCGTTGGGTGTGGGAATCACACCGGAACCCTTGTGATTGGAGG

AACGACAATCCACGTTGAGTGCGTGTTGAATCCGATTTCGCAAGGTTGGAGAATGGCCAAACATGTGGTT

GACAAATACCGGAGGTTTGGCACTGCTGGAGTAGGAGGTGTATGGCACGACTTTGTTGGAAAGTTTTCAT

TGTTCAGCCTGTTGTCCAGCTCGACGATTCTCGTTGGGCTAGCTGCTTTGGTGTTGCTCGACAAACGGAT

TGTTTTCTTGCTGCTGTTGTGCGGATACTTCATCTACACCAAGGCAGATGTGGGTTGCGGATTTGACCCC

GAGAGAAAGGTTGTGTCCTGCGGATCCGGAGGGTTTGTGTGGAAAAGCTTGTCTCAGTGGCCGACTCGAG

AACACTCTGTTGAACTCGATGACCAGCACTTGATCACGGCTCTTGTTTCGGAACAGTTGAAGAAGACGAA

CAAGGTGTGCATCATCTGTGAGGATGTCCTACAATGCGCCGCCGCTCGTGGTGCTGTTGGAGACATTACC

CACGTTGACAATGAAATTGTTTACGTTAACACTTCGTTGTCTTTTGACAGGACTTTTCCTCGAATTCCGA

AAAAAGTCCATGGCGTTAAGATCGGTGATCTAACAATGCAGCTAGCCCTAGCTTCAGTTGGGGGAGCGGT

CGATCCGAGCCAATATGGTGAGCTGAGTAGTGGGTTCTTGAGCCGCACCAAGATCGCGGAAACCGGTGAG

CACAAAGTCATCCGCGTGATAACTAGTGCCTCTCCATATGAAAAGATCTGCGAGCAAGCGTTCGCTCTGC

AGTACGGTTTCGTCCGGTTCACCCGCAGGGTGTTTGGCTCAAATGTCGTGGTCAAGCCCGTGGCAAAACC

GACAGATTACTGCCCCACGTACCTAGCAGGGTCGTTTGTGAAGAATGACATAGGCGCGTACACTGACGGG

ATGATGTGGATGAAGAGCAAGAAAGTGAACGGGACGTGGACTTTGGTTGACTTGGAGCTGACACAGAGCC

ACCAATGCATTTGGCCTCAAGCGTACACGTTTGATTTGACAGCCTTTAACGACTCGTCGCTGTTCATGCC

GGCGCAATACGGAGCTCCGATGTCCAAAGCCAACCACATACCGGGCTACAAGACGCAAACAGAGTTTCCT

TGGTACAAGGCAGATATCGTGTTGCGCGAGGGAGTGGTCCCTGGAACGCAAGTTGAAGAGAGCCCATCTT

GTGACAATCGCGGGTCCGCAGTGAAAGTTGATCCCGCTATCGCCAAGAAGTGGTGTTGTAAGACATGCCT

CTCAGCTGACAAACGGGTGTTTCACTTCAAGGTCGACAACGATTACTTCTACCCAATGGAGATTCGACCA

GCGGCGACCCAACCAGAAGTGACCATTGACGCTGACGGCGAAGACATGGACGAGATGGCATCAATGTTCG

GGACAATGAAAGCAGTCGTGCCGCCAGTGGAGGGGTCCTATCCGGATTTCCGGCTAAGCCCATCCGTGGA

GGGAGTTTCTCCCCTACTGGTGGGCGCGCTTTTGCATCTGCTGACCATCCGAACCAAACATCGGTGGGCC

CAACGCACATGTGGAACTTGGATTTTGTTCCTCCTGTTTGGAGTGCCATCAAACACCTATGCCGGGTGGT

CTTGGATTGGATTGTCGTACTCGCTCGCTGCTGTTCCGAATGGTTCAGCACTGTTGGTGCACTTTTGGCT

GGCAGTTCAGCTCTCGTCATCGCACTTGTTCTTTTTGGGATGGGCACTACGGCAACGTGTACGATCATCA

GTTGGGTACGCATTGAGCGTGTTCTTCGCCGAGTGGCTTCTCTTGAAGTTACGGCAGCTGTGGGAATCGA

CATACCTCCTCGACCACGTGCTTTTCCCAATGTACGTGATGTTGGCGTTCAATCTGAAGTCACAGTTCGT

GCCTGTGGACTCGATGGTACTGCTGAACTATGTTGTGACGCATCCTGCAGTTGCGACGGCCACGGTAACA

GGAGGTGCCCTGGTGATTTGTTCCATTCGCGTGTACAAGAACTGGGGATGCTCTCCGAACTTGTGGAGGA

GTGGACTTCGAGCTTCGAAGCCCTCATTAATAGCTGGAATGTGCTTAGCGGGATTGTACGTGCTCTCAAC

GTGTCTCGAGTTCTACCAGATGCCGACGACGGCGTCAGTGGTGTTCCTGGGGGGACTGCTGATCGGGATA

GTGACCAGGATGGCCCCTCCAACACACCTGGAGTTGGTTCCAGTGGCTGGAACAGGGGTGCCGTTGGACT

GCGAGGAAGAGCCCACAACACTTCCGTCTGGATTAGAAGGAACTTACGGTCCGGATGGAGTGGAATTCAC

GAACTTGACGGACAACAGTAGAGTATCAACGGGGTTGTTGGTTTACGTTGGCTGCATGGGAGTCATGGCC

ATGAACACCTACGTTGGAGTCATCCTGATGTGTGCTTGCTGGTGGACGAACGCTCCTGAGTGGCTACCTC

TTTACGTAGCTGGATCATCGATCTTTCGATCTAACGAAGTGAACGACGTTCTTATTACCCCTCCAGAGTA

CGAGCAAGAAGCGCAGTTAAGCAACGACTTCGGGCACCTGCCAGACGGAACATATCGGGTAGTCGCTCGC

TCACTACTTATGACCAGCCACGTGGGGGCAGGTTATGCCAAGGACGGTGTGTTCAACACGCTATGGCATG

TGACATCTGGTGGAAGTCTCACTTGGCAAGGGAGACACGTACGCCTACACTCCGGTGATGTTTACCGAGA

CATGGCTTCGTATGGTGGACCGTGGAACATCGCTGATTCACCTGAGAGTTCGGTGGTTGTCCGCGTTGTG

CAGAACGATGGTAGTGTGTTGTGTGCCAGAAGTTCTACGGCGAGCATTAGCGTTGATGGGAAGCGTGTCC

AGGTCATTGGCCATGACTATGGAAAAGGATCGTCAGGATCTCCGGTGCATGCGTTGGATGGCCGCGTTGT

TGGTCTGTATGGATATGGATTTTTCATCGGATGGAAGTATCACTCCTTAATCACAAGTGGAGAAGTCGTT

GCAGAGGACACTGTTGAGGAGGACACAGTTTCTCGGCGGTTCGTTGATTGGCACCCAGGAAAAGGAAAGA

CCAGACGGGTGTTGGTCGAAGAGGCTAAATCCCACATCGCAAAAGAGAAACGTCTGCTAATTCTCACGCC

CACTCGAGTTGTCAAGGATGAGGTTGTGCGGGCTATCGCTGAGGCGTGCCCTGGAGTCGTGGTAGGGTCA

AACCTAGCGATGTACCGGAGGAATGCCATCACGGTAGCTTGCCACGCGACGCTAACGCAGTATCTGATGG

AAAAAGGCATTGACAGCATTCGGTTCTCCACCATCATAATGGATGAATGCCATTTTCTGGACCCCCTGTC

CATCGCTTGCCGTGGGGTCATGGATTACTACAATGGGAAAGGAGTGGCCGTGGTGTACATGAGTGCGACC

CCCCCCGGATGTGCCGGAAACAACGGATCAAATCATCCCATCGCCGACGTAGCAACGCAATTTCCGCGTG

AGTTGACAGCATCGTGGGTTCGTGGACAAGCGAATGGCAAGACGATAATATTTGTTCCCACGCAGCACCA

GGCTAACACTTTGGCTAGTGAGCTTGGAGGAGTTTCACTAACCCGCGAATCGTTTGACGTGGCGATCGGG

AAGGCTCGCAGACAGGAGACACAGTTCATCGTGTCGACAGACATCAGTGAGATGGGAGCAAATCTCGGTG

TGCAGACGGTTATTGACACGCGTGTGGCAGTAAAACCTGTCATTAGCGAAGGTTCTGTTATGCTGGAGAA

AGTTGGCATCACACAAGCATCAGCTATCCAGCGCCGTGGAAGGACAGGACGCAGGGAGGCAGGAAAGTAC

GTTTACCCCATTGGGTCTGAGCTGGAGAACGAGGCCACTGAGTGGGCCTGCTGGACTGAAGCCCAAATGA

TCTTAGACCAGATGGCGTGCGGACCGATGCGTGAGGAGATCGAGAACTTCCAGCCACAGGGTACATATCT

CTTGGCACCTGAAAGCCGACCCCGGTTCATCAACTTCATCAAGAAAGATGTTCCAGTTTGGCTTGCATGG

CACTGGGCGAACGCGTTTGAGCATAAGCATTCAGTGCTGTTCCAAGGACAGAACGCGACGAGTTTAAAGA

TCAGGACAGAAGCTGGTGATCACAAGTACGCCCCGCGATTTCATGATGATCGTTTCGAAAAGAACAATGA

GTTGGACAAACGCTCCAAGATCATGCTGTACCTCAAACAGCGGAGCAACTTCAACTTCGATTTGGGGGGC

GTTCTGTATGGGCTGTTCGTGGCTTTTCGTGACACGAACCTGGAGCGACTTGGCACTTCTTACCGCTCGG

CGATTGAGATTCTCCATGAGATCTCCAACGTTGATGATCCAATGGTTTCAAACGTGGTGATGGGAAAGTC

ATTGCAAGCTTGGGCGGCCGTCATCATTGGGATGGTCACTGGCATCGTGCTGCTGGTTGTGTTTGTGGTG

GTTTGTCGCTGCGTGAAGAGGCTGTTCGGTGGAAAGGCCGCTGCACAGCAGAGCCCCCCGTACTCAAGCT

TCCCAACAGTACAAGCGGCCGGATTTTGCCAATTCGGTTCAATGGTGATGGCCATCGGACCACTGTGTGC

AGTGGTTGCCGGCATCCCCCCCGCCTTCGTTTTTGTTGCAGTGCTGGGGCTGTTCGTGATAATGTGTTGT

AGCGCGAACAACGTGCACCGGGCTTACACCACTGACACCGTCACGCTTGTTGTCATCGGCGTTTGCGTGT

GTGTCATGGGAGTTGTCGCATGGGAGATGGACCTCCTACCTAACATCCGACGGGACTTGGGTTACCTCTT

GGAAAGGTTCGCTGCAAAGCAAGAGCCGGACATCCCACAAGCATCCTTTGCACGACCAGAAGTTCCAGAG

TTGCACATAACGTCTCTTCCAGGGGCATTGGTTGTAAGCTTTGCCATCGCGATCGTTGGAGGGGCTATTG

CTAACTGTTTGTCCGACAGCGGGTTCTTGCGCAAGTTGTTTTCGAACGAAGAGCAGTCCGCCGCGGTAAT

CGGAGGCATTCAGCTCGCCTTGATTTCCTGGGAGACGATGGTGCCAGTTGCTTTTGCTGGATTCTTTGCA

ACAACGTTTGTGACGAAGATCTATGGATGCATGGTGGGAGGGATTTACCTAATTCTCGCCCACTATGATC

GGAAGTATGCCTTCACAGTGAAGGCGACTAAGGTCCTCATCGCTCGTACCAGCAGGAAAGATCTCGACGA

TGAGATAACAGGGAGAGACGGAGTCACGCGGGGCCGGCCAACTTTCTACGCTCTGCAAATATGTTGTTCC

TTGTTGTGGACAGTCACATCGCCTAGTCTGAAGCATGTGGTCGTGAGCGTAGCGGTGATCGTGTTTGCAT

TCCTCACATTTCGTCGTCCAAACAACCGGCTGCTCGTCACGTTTGATTATTCGAGTGTGTTGTTGATCCT

GATGATCTTCGCTGAACCTGGGCAGGTTTTTCTAGTGGGTGCCAGTCTACTGTTTTGGTTCGTGGCGCAC

CAGTCACGCATGGCACTACGATCGTTGGTGAAGACGGACGCTTGCGGACTGGGGTACAGATGGAAGGAGA

TGCTCAACGCTCTGGACAAGAATGCGTTTGACAAGTACCGCTCCCGGGGGGTGAATGAGACCGACAAGGG

TGATTTCGTTTCGCGAGGGGGCTTGAAGATGGATGAGCTCATCAGGAAGTTCCAATGGGAACCAAGGGGA

GCCGCGCTTGACTTGGGGTGTGGCCGTGGAGGATGGACCCAGCGATTGGTAATGGACACTAGGGTCAACT

CCGTGACGGGGTTGACCCTGGGCGGCGCGAGCCGGGAGAATCCCGTACCGTTCAAAACGAAGGGGCACAA

CTTAGCTGTGTTGAAAGCCGGTGTTGACGTGTACGCGCTTGAGCCGCGAGATTGCAACACGATCGTGTGT

GACATAGGTGAGAGTGATCCCCGACCGGAGGTTGAGAAGACAAGAACGCTGAAAGTGCTAACGATGCTTG

AAAAGTGGTTAATCCACAACCCAGGGGCTGCGTTCTGTTGTAAGGTGTTGTCTCCGTACCACCTTGAAGT

ATTGCGCAAATTGGAAATGCTTCAACATAAGCACGATGGAAAGTTGGTTAGGTTGTCCTTGAGTCGGAAC

TCCACTGCTGAAATGTACTACGTCTCAGGTCCGCGAGCAAACATCGTTGGTTCAGTTTTCCACGTTTTAG

GAGCACTAATTGGGCGATTCAAACGTAATGATCCGGTGCAGCGAGACGCTCCTCCAAAGCTCGAGATGGG

CACTCGAAGCGATCCCCGGGCAAAAGTGAAGCTGCAAGACCCAACAATCGTTGCAGGTCGCGTGAAGAGG

TTGCGAGAGGAAAACGCCAGCACATGGTTCGTAGATCGTGAGCACCCTTATCAATCGTTCAACTATCACG

GGTCATTTGTCACTGACGACATCTCTCCAGGAGGGCAGACGGTCAACCCGATGATGCGCCGAATAATGTG

GCCGTGGGATTTTCTCTCTCGTGTGACGACGTTCATGATGACAGATGTGTCAACGTACGCACAGCAGAAG

ATTCTTCGTGAAAAGGTTGACACGCTGACGCTTGAACCAGACCAGCGGACTCGGGCTATCAATCGGTTGA

TCATGCGGCATTTCTCCGCGATGTTCAAACGGCGCGGATTGGCCCCACGAATTCTCACTCCGGCGGAGTA

CATGAGTAACGTGAAAAGCGGAGCTGCCATTGGAGGGTGGAGCAAGGAGATGCCATGGAACAAGGTGCAG

GAAGCTTTGGCTGACCCTGTCTTTTGGCGCATGGTGGCTGACGAGCGAGCCCGGCATCTGCGTGGAGACT

GTGAGTTGTGTGTGTTCAACACAATGGGCAAGAAGGAGAAGAAGCCCTCCTCTTTCGGCGAGGCGCGAGG

ATCACGGATTATCTGGTACATGTGGCTCGGAAGCCGGTTTTTGGAGTACGAAGCACTTGGATTTCTCAAC

GAAGACCATTGGGTAGCGAGGGAAAACTTCCCGTGCGGAGTTGGCGGGGTCGGAGTGAACTACTTTGGAT

ACTACCTCAAGGAAATCATGCAAAAAGGGAAGTGGATGATTGCGGATGATGTTGCCGGGTGGGACACGCG

CATAACAGAAGCTGACTTGGAGGACGAGTTGTGGTTCCTGCTGGACCAAGTGAACGATCCTTACCACGCC

AAGTTGATTCGCGTTGTGTTTAAGTTTTGCTACATGAACATGGTCGCGCTATTTCCAAGGAACCATCCGC

AGTTCCGAAGCGGAACAGTTTTTGACGTCGTTTCACGGACGGACCAACGCGGGTCTGGGCAAGTGACCAC

TTACGCTCTCAACACTGTGACGAATGGGAAGAACCAGGTTGGGAGAATGTTGGAAGCAGAAGGGCTACTG

GATGCACCGTTGGAAGTGATCGATGGTTGGCTAAGTTCCCACCTTGAAGACGTCTTGAGTGGAATGGTTG

TGGCTGGGGATGATGTGGTTGTGGCAACCAACAACGAAAATTTTCACACCAGTCTTCGGTACATCACTGC

TGCGTCAAAAACGCGCAAGAACTTGCAACCTACCGAACCGTCCCCGAGGTATACAAGCTGGGAACATGTC

GAATTCTGTTCCCACCACTACCATCCTCTCGTGCTGCAGGACGGTCGTGAGATCATTGCCCCGTGTCGTG

ACCAACACGAGATTATCGGTCGGGCGCGGATCCAGAAAGGAGGAATCGTGGACATGTCCGCAGCCGGGTG

CCTAGCGAAGGCTCACGCCCAGATGTGGGCGTTATACTACTTCCATCGGAGAGATCTGCGACTCGGATTC

GCCGCCATCACGTCGGCCGTCCCGGTGAATTGGATCCCCACGGGGCGGATATCGTGGTCGGTTCACCAAC

ACGCTGAATGGATGACGACACAGGACATGCTAGAGGTGTGGAACACGGTGTGGATTGTCAACAACCCATG

GATGGCGACGAAGGAACTGGTGAAGGCTTGGTCAGAGATACCATACCTCCCGAAGACGAAGGACATCAAC

TGTGGAAGTTTGATCGGTGAGCGGGATAGGGCAGCGTGGTCAAAGAATATCGTGGCCACCGTCAGCACAA

CCCGGCGAATCATCGAGCAAGAAGCAGGAAGCCAAAAGTTCACGGAAGGGCTGCGGATACTTGGGCGATA

CCGAGCTCCAGCCGACGACGTGTTCTGGTGAGGATCACGCAGGTCGTAGAGAAATACCTCTCTAGAAACG

GTTAACGTTGCGAAGCAACGGGAACCCCGCAAGGAAGGACTAGGCTGTCCTTGGGTGCTAACGACACTCC

GGCCCCAGTTCCCAGAGCCAGGGTTTTGGCTTCACGGTGTTGGAAGACACCCTCGCGGCCATGGCTGCAC

AATGCGCGCAAGGAAGGACATGGCTGTCCTTGGGTACTAACGACACCCCGCCCCCAGTTCTCAAGGTTGG

AGCCACAACCTCAGGATGTTGGAAGACATCCAGGCCAGAGCAGGGCCATCGCAAGGGAGGATTTTCCTCG

GGTACTGACCACACCCCGACCCCAGTCCAATAGGTCATGGAATGGCCCCGTGGTGCTGAAAGGGCATCCA

AACAAGCTGAGCATCTTGGAGTCTGCTCCCGTAAGGAAAGCGCAAGCTTTGAGCATTGACGACGCTCCGG

**>OK413943.1 MAG: Hubei chryso-like virus 1 isolate Mygg_341 segment 1 RNA-dependent RNA polymerase gene, complete cds**

GAAAGATATAATAAACGATCAGTCTCTCTTATCCGCCTTTCGAGTACGAAATTTTTACTTACTTGATTAC

CACTACAGACATTGTGACAGAAAGAACTGAGAACTCTTGGTTAGAGAGGAGAATACTGCAGCGGCGTGTT

GATCTGCGATGGAATTGGATTTCAGCATATTGAAGAACGCTTCTGGAGTTAAGTTGAGGAAAAGATATTG

TGTCACTTTGGACGATTCACCTTGTTCTTTTGATTCAAACTCGTTTGACTGTAGTTTTGGTTTTGGACGT

TTGGGTTCCACGAGTTTCGCCATGCTATCAGATAGATACCGAGCTGCTGAGGTACCTGGCCTATACGCGA

TAGTAATACCAGCGGGGGGTGGGAAAACGACTCTCGCCAAATTTTTCAAGCAGTTGGATGTTGATAAGGT

ATTACCTCCTGCGTTTGAGACTGAGCTGAGAAAGATAAGGATGAGTACATTCAACTCTGTATCCGGAGGT

GGTACACGGGCTTCCTGGTTGAGGCATAACTCAATTTGGGCTAGGTATTTGAGCCTATCCTTGGGTAGCT

TTGATTTCCGTGAGACACCGAGGATACTATTCATTCACTCTCATGAGATAGCCACCGTTATCGGTGCTAA

GGTAGTGGGTATCCTATTACCTGAAGAAGGGCTTCACGAGAGATGGGTGTCAGATAGGGACCATGAGGCA

AAGGCTTTAAGTTACGAAAACAGGGAGTTTCTCTTCCGATACGCCAAGAGCCTACCTAATATGTTTATCT

ATGGTAGTAGCCTTGACCTTTATAGGTTGGTTTCTAACATCATCGTGAACACAATAGGTTACGCACCAGG

TGTGTCAGAGTACCTCAATGTGTCCGAGATAGACATGGTGATGCTAAAGCAAGGATATGATATTACTATA

CCCACTAGGATTAAGGTGGGGAAGTTTGATTATGATGACCTGGATAGCATCATTGATTGGTGCAAAGCTG

AGAGGTGCCCGTGGTGGTATGTAAGTGTGTGGTGTGAGAGGTACTCTCCGGGGTTGATAGCCGATGGAGC

TTATTCACTTGAATCCTATCCATGGTTGCACTTATGTTACTCAATAAATACAATACTGAGGAATAGGCGC

GTATCCTGTGATATATCCAAGGCCATGGTCAAAACAAATCTTGACTGGTTTGGTTTCTTTCCACATGTGG

ATACATTAACGGAGTCCAGAGCGGGGGTATCCCTTAGATCAGTATTCAAATATTTAGACCCGGTGTACAT

CGATGAGTACCTGATCTTAATGTTGAATTGTCATGTTGGCAGCCACCACTCTTTTGTTACTTCTCTGGTC

GTATATTACTTGGGTGTGATACAACCTATGCGAGCCGAATTGAGGAACAAAGTATTAGACTGTGGCATGC

TGTTAATTCCAGAAGAGCATTGGGTGTCAGTTCATGGTGATATACACAAGTTAGTGAGAGCTAGTCAGTT

ATTCTTTGGCTTGAGTCTGAAGGACAGGGAATATGCGAGCCTGCAATACACAGCTTCGTTATATGGTAGA

AGGAACTACTCTCTAGATCCCGAAGCCGAGATAGTTAAGAGGCAACAAACAAGATTGGATACAAAATGCG

CTCAATTGCACGATGGTGTGAACAATGAGGTTTATATAGAGGACTTCAAGGCTGGCGTTCGCCTAGCGTA

TAGCAGATTGGGTAAAAGAAGCAAGTTGCGTTGGAACAACTTTGGTGAATTCTATCAGCAAAGGTACCAG

TGGGCAGCAGCTGGCAGTGTCACGAACGTACCTCCAAGCATGCGTAAATTCAAAGAAGTGACTGAATTGC

TCGCTGAGGTAAGGGGTCAGATTGTGCACCTATCTATGGATAGCAATAAAAAGAGGGCTATGGAAAAGCT

GAGTGGACCAGCTGAGTTAGCAAGATACTTAAGTGATAATTGGGCTTATAATGTAACGTCCCTCGCCCCT

AAACCCAACGAACCGGCGAAGAATAGAGTTCTAATGCCTGGTTCATTCCTACATTATGTAGCGATGTCTT

ATATACTGGGGATGGTAGAAAGGACTGGGGATGTTGGAGCAGTCAGGGTTGGGGATCCGGATGATAACAA

TCTGAGCCACTTTGACTTACGATTGACATCCGGGACCTACAATTTTATGCTTGATTTCGCCGACCACAAC

GCTCAACACTCTAGTCTCGAAATGGGATTAATCATAGCCTTACTTGAAGAAAAATTCGCTAATAAATCGG

ATGCTTCGGATCTGAATTTCTTTATCAACTGGGTCGTCGATTCATTTGTGAACATGCAGATTAGGGTGGG

ATTAGATAACCATAAGGTGATATCAGGATTATTCACCGGTTGGCGTGGTACGACCTGGATAAACTCTGTG

GCATGCCAGGCATATGTATACGTCGGGACGCAGGCTTGTAAAAGGAAGTACGGATCAATAGAGGTTGAGT

ATTTTGAAGGAGCTGGTGATGATGTGTTAATGAAGTTTAATTCGGCGAAGGATGCTTTCAGATTTTATGG

TGCAATGCAAGCTTGTGGATTTGATATGCAGTCAGTTAAACAAATGGCCTCACACCGGAGGACCGAATTC

CTTCGCACGATAAGCAGTAATGGGCACTTAGTGTGCTGTGTGAACAGAGTTTTGCCCAACTTTATTTGTG

GTGATTTGGAGCGATCTAGTGACGGTATGGTAGACAGACTGGGTGGTTGTTACGCTACAGTCAAAATGCT

ATTTCGTCGTGGATTGTCCGAGCATATCACTAAAGTAATTTATAAGAGTTATCTAGACAAGTGGGCTAGA

GTAAAAGATGGTGATGTGTATAGGGACATAGACAGGACTTATCTACACGCACCAACCGAGCAGGGCGGAA

TTGGGCTACCCGACGCCGATGATAACTTATGGTACCTGGATAATCCGATCAGCTTGAAGACGTACAAAAC

GCGGGTGGTGCGGGGACCTAATAATGCTTCTTTGGATTACGCCAGACACTTAAAGACCGACTTAGCAAGC

AAGGGCCTGCATGTGCATGAGGGTAGGTTATTGGAGAGATTGATGTCGGATGTGTATAGTACTGAGAACC

AAGTTGAAATCGGTGATATAGTAAATGTTGGTAGCAAAGTATTGGCATTGATCAGTCCAACGACGAAGGT

GGACAAAGCTGTACTAGATGAGGTTCTCGCTAATGTGGACGATGAGAGAGTAAGAGAGTACAAGAAACAA

TGGGGATTGTACAACAAGTATAAGCAGTGCATTAGTTGTGTTGAGGAAAGTTTAGAAGTATTGCTCGATA

AACTGGGAATCAATATTGACGTGGTAGCTTTAGAGGAGCTTAGGTTTGCGAGCAACCACTGTTTCTTAGT

GCCTGAGTACATATTATACAATATTGGTACATATTATAGATCAAGGGTGGCTTTCCGGATGATGTCGATT

ACGGAGGCACAATACTACTTCAATGTAGCCTGCAGCACTGCCAAAGCAGCCTTTGGGGAGGATTTGATGC

TGTGAGAGAAATATTGAGGAATGTTATTTAATTAGTAAAATTATTTCCGCCTATTACAGATGGTGATGTG

GGATAGTTTTACTAATTAAATAACATTCCTC

**>OK413944.1 MAG: Hubei chryso-like virus 1 isolate Mygg_341 segment 2 putative protease gene, complete cds**

AAGATATAATAAACGATCGGTCTCTCTTACAATCAAGTAAAACAGGAGGAATCCTTAATTATTTTGTTGC

CACTACGAACGAGATTAGAACAAAGTGATCTTCGGTAAGGCGACGAACTCTAGTGAGCGATTGTCGATTG

GTTAAAGGCTCGAGTGAGTTAAGCGGCTTACGACGGTTTCTGTCTTACCATAACCTGCCCAATATATTGC

CAGGTTAAACGAGGAGTGTGTTAGTTCCAAGATGGTTATCAACACATCGCATTCTGAGTATATGAACGGT

AAGGACAAGAGGGAGGTCCATCGTAATAAGTTGGCAGCGTACAACCCTGATAGCGTGAGTGATGCCACTG

TTGATAACTTAGATCAGCACGTCGCTCGTAATGCATCAAGCATGGGCATAGGGATCAAATCTAATAGTCG

TCAAGAACTGAACCAGCGTCCTCCATCTGATGGGATTCAAGAACCGGCGGCCGGTGTTAGTGGTTTTCGG

CGGTTTCGGATGCCGATAAGTGAAAGGAGGAATGAGACTGCTAGCGTGGGTTTGTCGCAGCTAAATGAGT

TGATGGGCAAGAACAGCGCTGAGGGCATATTTGACCCTAACGACTTGGATTACCTGTTGTGGCATAATGA

GCCGAAAGAGGTTGCCACTAGTGTGCACTTCGCGCAGATGCTTAACTTTGAAGGTGTAGGCGAAACGCTA

AGAGTCACGTTCAGCGCCATAGCAGAGGATGATGACAATAGAGCTAATAATTTTCTGACCGAGTATATAA

TGGAATACCCAATATTCAAGGACCCCACTAATAAGGTGAGGATGTTATCTAATGCTAACGGAAAGGAGGG

ACCTAAGACGGGTATGGTTAAGTTGATTAGCCAAGCCACGCACGCAACAGGGCTGTGTGAGGCCGTAGCA

GGCAAGGGAGTTGGTGTTGAAATGAGTTTGTTGTCCGGGAAGAATGTCACCAAGATGCATACGATTAAGT

CTAAACCCGTAGACATGCGGCAGATGGCGCTAAAGGCTGTAATGCTCAAGTTGCAGTTGACATTGTTGCA

TTCGGCGGAGGAAGGAGGTGTTGAAGTTGAGGCTCGATCGCTTAAGAAAGAAGTGCCTGTACTGCGCACG

TCGGAGGCCTTGGACTTGTTCCCACGGCATGATGTTATCGTGGATATCACAAAATTCAGCATGGATCAGA

GGAAATTACTACTGGTGTTGTGTTCAGCATGGCCTTCACAGAAGCTGATCAACGGTGATGAGCAGGACAT

ATACAGCCTAATAACTTTTGAGGAGGAGAACTTCTCTTTCTATGTGACCAGCGGAGAACCTATGACTCTA

ACAACTGATGGTTATCTGTTAACGCCGAGGGAGCTGTGGTCGCAGTGTGTGCAGCTATTTATGAACATGG

GTGGTTTTGATGATTTGGTCACTGTTGTGAGGGATAGTAGGGGCCTCGCCCCCATTCTGACGCATAACGC

GTCCGCTGCGAATAATAATATTAACGTCGTGTCGGCTTACCCGTCCTCAACGTGTTACCATGGGTTGAGT

GTTAATGCCTCACCTGAGAAGAGGTACATTGCGCCTACTATCCTGGAGTCCTCCAGTCTCGTGCTACTAG

TGGACAATATCATGTTAAGCGTGTACCTGAATAACATGTTGTATCTGGCGGAGGAGTTGGGTTTGGGTAG

CACAATGCTTTATCCCCGTCCGGATGCTAGGGATAATGGGAAGATAGCTGATGTGTTATATTCCCATGGT

CTAAAAGGCAATTCACCGCAAAATAGTCTAATGGGCAAGGTATGTCCTTGGCTAGCAAAGTTGGACAGAT

TCCAGAACGCTGGTTTGCATGTGATGTTGGACATCGTGAATTCGATGCGAAGGGGTGGTGATGCAGGTCT

TATCTACTGCCCCCTAAATTACGCGGTTTCGACATTGCAGACAGTGTGCACAGCGTCTCTAATGAAAGAG

AGGATGGAGATAGATTTATCGAAGGTGATGGGATTTAAGTCCAATGTTTCTAAAACATGGGAGGTGATTA

AGTTACTCAATTGGTACAAGGCATGCTCAGGCGCCTCGCTGCCTTGCTATGGCACCAGCGTCTTCGGTGC

GAAACTTAAAGGTGACGAGATGAGGTTACTGGGGGACGTTATCCATAAGAGGGGATTCTTTAAAGTTAAT

AGAGTCATGGCGGTGACGGACTATCTGCCGCAGAGCGCTGAGTCTGGCACTCTACAACAAACATTCTTCA

ATAACCCAGGGTACATGGCGACAGAGCAGTTTCAGGCGCAGGAGTTGTACCAGGTGACATACAAACCGAA

TAGTTTTTCATTAATGACCGCTTCTGGTGAGGCAGATAAGAATAAGTTTCAGGAATTATATAAGCAGAAC

GGTAAAGCGGGGAGCACAGGAGGAAATGCCGTTGGAGGCCCAGGCCCGCAACCAATCCAACCGGGAGACG

GAGGAGGTCAGCAGGAGAAGAGTGGAGTGGAGAAGCCGAATGGTAATCCACCGCAGGGTATGCTAACATG

TATGGAGATACCCAATGTGGTAGAAAAAATAATAGAATACACCCCAGGGGACTGCGGGATAGACGCACTG

GTTAGTCTGATACCTAGTATCCGAAAGGGCGAGGGACTCGCCAAATTGAACTTGAAAGAAGGTGAGAGAT

GTTGGCTAACGGCTGACGAGTTAGGTAAAGTGGCGCACGAGTACGACACAGACCTGATAGTGTTGAAGGA

CTCTGGTCCGCCAGAGTACTATGCTTCCAGCAGAGGAATGAGAAAGAACCAGGTGGTGGTTAAGCAAAAC

GGACACCACTTTGTGCCTTGCATACCGACCGGAGGTAAAATAAATAAACCTACTCTAGTAGTGCAACTGG

AGCCGCTACCGGTGGATGAACAGGGCAGAGAGAGCATGCGTAGGAGGATAATAGCTGGTCAGATGGTAAC

ACCCGGCTCAATAACTGATGATAACGCTGAGCGCGGTGTGGTCCGTCGGACTATTGGAGATGGGACTCAG

GTGACTTGGGGATCTAGTGCGGGATCGACGAGCAGTGAAGATATGACGGTTGTGACAGTCGGCGCTCAAG

CTACCAGGAAATGATTGTAATAGACCAGCATTTGAGTACTATTTGGAAGGAGAAATCGCCTGGGGCAAAG

GAGATTACTGCGATACGAGCTGAGCTTAATTAGTGAGCACAGTGGGAGC

**>OK413945.1 MAG: Hubei chryso-like virus 1 isolate Mygg_341 segment 3 hypothetical protein gene, complete cds**

AAGATATGTTTAGCGATCTGGCTCTCTTACCACGAATAGAATAGTGAAAGAACAAAGTAGTGAGAATCTT

TTTCCAGGATTGGTACCTGCAAGCGGCCAAAGCTTGACAACACGAATCGGAACACATTGTTCAAATCTAA

CTTGTATTCCGAAAGGCGCAGAGTATTGGTGTAGTGCTCACGGTTTGAGCGGGAATCAGCTGTTAGTGTG

GGATCAAGATGAGTATGTCGAGGAGAACTGTTCCGTGGGTTGAAGGTGATGATCTGGTTCCAATGGATTG

GAACGTGATACATCCATTGACCCGTCAGAGGATAAGTGTTAATTTGACCAAACCGACTAATATGTCAAAG

GTTGAATTCTTCGAGTTATTGGTTAAGACTGGTACCACTGACGAGTTTTTGACAATTGGCGATGATGAGT

TGTCTTTGCTCGTGGCTATGTCTAATCTACGGCGAAGTAGAATGGATAATTTAGCTAAGCAGGAGTACAT

CGACTCTAGAGCAAAGAAAAGAATAAGTGATCCGTTCCCAGGGACGCCTGAGTTATGGGAATTGAGTGTG

CCAGCTAGATCAGTTTCTCAGCGGGCGCTACACGAGTGGGACCCAGAGTATGACGAGGGTACAGGATCAA

GTCTAAGAGCTCACGGTAAGCACCTCAATGATAATATTGTGATGTCCGGGTTTTGGGGTAAGGATGATAA

AGTTTACAAGTACAAGTTGTATATAGAGGGTGGTTTTTATGTCACCATCAGTGCAAAGGAGCCTTTTGAT

GATTTGGAGTTAATGCAAGCCAAGGAACAGGTAAGGGTGATGTTAACGGACCCGCTTCTGGAGGAGGAAT

TGGTCATGCAGAAGGGTATGAAAGATACTAATCTAGAACTACCCTCGACTCAATCGGCTCTGAAGGATGG

TTATAAACTACTTGAGTGGCTTAGAGTAGGAGATCAACTCGCCAAGGCTTATGACCACGAGGTGGTTGTG

AAGGACATAATAGAGGGTGTTCCTCCCCCCTACACAATGCAACTGCTGCAGGAGTTCCCTTCGCCCACAA

GCACCGTGAGGTATGATTATGAATTCTCACCAAGGATACGTGAACCCTGGATGGCAGATTGTCATGCCGG

TGTACTGCCGACCTTTGACATGGAGAGCTTCTCTAAAAAAGTGGGACCAGTTTTACCGATCGTTAGGTAT

CCTGATTTGCCGATTAGTGACATCTCTGGTGACACGGTTGAGTTGAGAGGAAAACGGTTTCATATAGTGT

TTGATCAAATGTCGGGCGTTGTAGACAAGATAACGCCTTTAAAGGAGAAATATAAGCTGGAGGAAGTGCG

TAAGAAAGTCAAGGTGGATAACGTGTTGCTGACTACCAATTACGCTCAACCCAAAAGACCCGGTTTGAAA

ACCAAGTACGTGATGGGCAGTGGTAGAAGGCTGGTTGTTGAGATGGGGCAAAAACATATGGTCCCAATAC

TGTACACAATATTGTTGTGTACTAGTATAATACCAAATGTAAGAACCCAGTTCATATGTGGAGATGACAA

GCATGGTACTTTATGGACCATGCCCGATCATGAGGATTGTAAACCAGTTGAAAATCGGGGTTTTGAGATG

GAGTTAAGAGTGTACAATAAACTATATAGTTATAATATGACGGTATATAGGTGTTACCGAACGGTCAGGA

AAGATACGACTATATATGGCTTCTTTGGACCTAAGTCGCACCTGGGATATACCATTAATCAACAAGCTTT

AGCTTTGGCAGATTGCGCAGGAATGGTGCATACCAAAGCTATAAGGAATCATCGGTTAAGCAGGGTTGGA

GATAATTCATGGAGTACGCAAGTTAAGCCCGACATAAAGTATGTTTGGTGTTGCTCAGAGCAGGTGACGG

AGACTCTGAATTATCACCTGGAGGAGGTACAGGCTAGGGTTCATCTGATTAATGGGTATGCCGTAATAGT

GAGTTCTGACTCTGACGTGGCTCATTGTAACTATAATGCAGGTAATTGCACTAGCACCACTAGTACTTAT

ATCTGGGAGAAGAAGGATGTGACCTGCGATATACAAAAAGTGGGTCAAGGCACTTTCACAGTTATAACTG

ACGGTCACGCAATAAGCTATGATCTCCAATTATCAATAATGATAGGCGGACATGTTGATTATTGTGGCGT

GATCTACAATACTACCGAGCAGGGTCTATTAGTACAGGAGATCTCAAGGAAAAAGAGGGATGTGGATGCT

GCGGAGAAAAATTACATATTGAAGGCAGCGAATGGATACACCAGAGAGATGGGAATGGGTCTATTTTACT

CGCTCTGCAACTTTGAGAGGATGTGGGTGAACTACATGACTGACATGGCTAAGTCGAACCCAACTAGATT

CGCCAGGACGTACCTGAATAGGCACGACATAGCGGCGAAGTTTGTCGGGGAAGCGTTGTTAGTTTGGGAA

TGCAAGCAAGTGGAAATCACGACCAAATATAAGGGACACAGGTTGAATAGCCAGTGTTATGACATGATGC

CAGTTGAGTATGAGATAGATAGAGTTAAGAAGATCGGCTTCTGGGATTCATCAACTAATGAGATATCTCC

TATCAGCAGCACACATGATTGTAATTCTGTTAGTGGTTACATCTTATTTGAGGGAGAATGGCGCAAGTAC

TTTAACAATACACATTACGACGTAGTAATAGCGATACAGAAGATGCCGAGTAACGTTGGAGCTATGGATG

CCAGAGAAGTATCATTCAAGAATAATAAACTCCACAGCGTGTATGGTCCAGCAGGATCTCAGTACCGCGA

GTTCTTGGTTAATGATTTAACGGCACTATCAGCCAAACATCTATTCGGTCATGATGTAGAGTGTGATGAG

CAGATTAGCACGAGGATAAGTGGGATGATCCATGACACGGCTGCGGGTATAAGATCAGCTCTATTGTGGC

CACTCAAGGCAATAGTGTTGGCCATGATTACGTTGGTGTCTGCTTATATACTAATCACAAGGTGCAGCTG

CCGGCGATGCTGGTCATGTACAAAGCAGAGATACAATCGCGTAGTATTCCGCAAGGATGGCGAAGTGGTA

GAAGTCCAGGAATAGGTGATTAAGCGCGGTTAACTCACGCCCGTGTGGTCACCTATTCCTGGACTTCTAC

CA

**>OK413946.1 MAG: Hubei chryso-like virus 1 isolate Mygg_341 segment 4 hypothetical protein gene, complete cds**

ACATGGATACGCGGAAGTATTTCATGGAAAACCGGTACACCCGGATGGGTGATATAATAGGTGAAGGTAA

GCGGGCGCTGGATACTCCCCGCTTTGAAGGCATGACGCTGGGGGATGTTTCACCATCTATGTTGGTTGAG

ACTAAGCCAATTAAACACCACGTTATCGGTAAGGCTGACAAGGACAAGCTCCTGGATTTGCAAAGGAAGA

CATTGTTAAGCATCGGTGCTAATAAGGATGGCCTGGCGCCTAAGGCTGGCGAAGTCTACCAAGCTATTAG

AGAAGCGGGTGCGGCTTACTCTAATGCCAGGAGTTTGGTGAGTATTGGTCAAATAACTAATGAGCCGGGT

AAGAACATGGTCTGTAGGAAGAAAGATTATTATGAATCAATCCATCATTTGGGAGCGCATTTTTTCAGCG

CTAAGCCTGCTAAGCTAAGGGGAGCGGATCCCTTTGTGTTCAGGATTGAGTATTGCATCTTTGGTGACTT

GTCTGACGGAGTTTGTGACGGTGTGGGTTCTGGGCAGTACTCAGTGGAAGCTAAATTAGAGATCCTTGCT

CCCACTGGGGGCGGTAATCCCAGGTATGCTACCAAATGGGTTGACTGGAATGATAATCCGACACGTTACT

TCAATGCCAAGTGTCAGGACGTGATAAGCGACTCGAGCTTAAAAGTTGAGGATTTAATGACAGCGCTGGC

TTTACCACGGGAAAATATTACGATACGCAATGATGAGATGGCTGAATTGAAGGCCTACTTAAAATTGAAC

CCCACTGCGTGCCTAGCTTCTAGGTTCATGGTACTTTACCTCGTAATGATGCGGCTGTGGCATGCTAATA

GGCGCAGTTTCAAGTTGGATTCATCAACGCTGAAGATACAAGGGGTGCCTTTTAGTGCACAGGGTTTGAA

TACCTGCTTGCGCGCTCCCTCAGGGAGTACAGTTATAAATGTGGTTAACATGACAGAGAAGGAGCAAGTT

ATCATGGCTCGTAGTGCCATGGGCTACGCTAACGGAGATATCCCGATCGACGGTTTCGACCTGAATATGG

GCGGCTTTGATGTGCAACCAGAGGTTGACAAGAATGGCATCATATTCTTATTGGGGGCAGAACCTACGGT

TAAGTACGCAGAGGAGATAACGCTAGATGATATATACGAAACTATGGTGAAGTACGCAATGAAGATGCAA

GCATGCGGTGAAATGGAGATGGGCTTCAATCTGGCTGGGAACTTGTTATTTGCTGACGGGTTACCAAGTT

TTAGTGTACCCCGACCCCAAGGATACGTGGACATAGTGGCATCAGCCCTGTCAAATGTGAACAATAGTGG

TTTTGTCCCGCCATACGCCATAGATGAGTTGAATATGTTGGGTTTCTTTGTCTTGTCGAGGCAACAACTA

TTAATGATACATGACGTATTGATGTATGCTGAAGGTCCGAAGGGCAGGGATGCTATACCATTGTTGCGGA

TGCCGTCTGAATCACGACAGCTGATATACCATAAGTACAAGGAGACGCGATTCTCTGACGCATTTGGGCT

GACGAGTTTAATTGACCCATTGCTAGTGCAGACCGCTGAACAAGTAGAGAGGGTGAGAGCTGTTGCCTTC

CCAACTATCGCATGGTCGTCGATCAATCATGACCACTTGGTGAAGAACACTCTGTCTGAGTTCTTTTGGC

GCGGACAATGGGTGGATTTGGTTGATTGGCGTAATCTTGATGACCAGTCTCAGCGCAATGTGCTGTCCAC

ATACTATGCTAATGGTTTGTTAAATGAGCCGAAGACTCTAGGTGTTAGAGTAAAAGAGGTGATTGGCGTT

TCACTGCACTCACGCCAGATGAGAGTGCCTACAGGTCCAGTATGCACAACGCTGGTATCAGTGGAGTGCG

TGCCGAGGTGCAAACGAGAGAGTCAGCAAAAATTCGCGAAAGTGCCTTTTAGTAAGCCGAAAGTGGTGGA

TGAGGACGAATCAATTGATACCTGCTTCACGGAGACCCCAATACTAGTGGAGAATGATGGGGAGAGTGGA

GAAAAAGAGGAAAATAGTGGTGGTAATGGCGCTGGTGCTAAGGAGAAAGAGACAGCAGGTGAGCAAATAA

TCAATACTGCGAGAGCCAATATTGAGAGTGTTGTCGCTAAAGTAAGCGATATAAAATTACGTGTTATTGC

CAAGCAGACAGAGGATCCCCTAATGGAATCGATAATTACGGGTATTGGGAAATACTTGAATAAGAAGCTC

TGCTCTGAGGACAGTCTGGTGCCTAGGATTCGACACTTGATGTATAGCTGGATAGAGGATGGAGTTGGGG

TCAGGTGCCCATTGCGAGGACTACTTGGTATGGGTGGAATGAGAAGTAAGGATGCCAAGCACTGGCCACT

TGTGGTCGAACTAATGTACCCGTACCTAATGCAACTGGACGAAAAAAGAGTAGTAAGTATGCATGATGGG

TTTAAATTGTTGACACACATTAAGGCCATTAGGAAGTACGGTTGTGCAAAGTCACACTTTGACATGAATG

CCCTACCCGTGTATAGACAAGATGGCGAATTGTACAGATGTTGGATAATTTGGTTGGGTTACATGGGTAT

AACTGTGTCATACAAAGGAAATAAAATGCCTGTTAAGGGTAGCTTCAGTGATAAGGAGAAAGATCCGTTG

TTGGATATGCTGATGGATATGACACTCTGGCTTAGATTCCCCATGGCTGAGAACCTTATGCATACCTGGG

GTTGGGAGCCTCAGCAGGTGAGTGTGGAGAGGACAGTGGACAAGGCATTCCTGGCAAAGATGAAAAGTGG

ACTAGATAAGAAGTGCCATGTATGCGTGTGTTAA
